# The contribution of non-malarial febrile illness co-infections to *Plasmodium falciparum* case counts in health facilities in sub-Saharan Africa

**DOI:** 10.1186/s12936-019-2830-y

**Published:** 2019-06-11

**Authors:** Ursula Dalrymple, Ewan Cameron, Rohan Arambepola, Katherine E. Battle, Elisabeth G. Chestnutt, Suzanne H. Keddie, Katherine A. Twohig, Daniel A. Pfeffer, Harry S. Gibson, Daniel J. Weiss, Samir Bhatt, Peter W. Gething

**Affiliations:** 1 Big Data Institute, Li Ka Shing Centre for Health Information and Discovery, University of Oxford, Oxford, OX3 7LF UK; 2 Department of Zoology, University of Oxford, Zoology Research and Administration Building, 11a Mansfield Road, Oxford, OX1 3SZ UK; 3 Global and Tropical Health Division, Menzies School of Health Research and Charles Darwin University, Darwin, Australia; 4 School of Public Health, Faculty of Medicine, Imperial College London, St Mary’s Hospital, Norfolk Place, London, W2 1PG UK

**Keywords:** Malaria-attributable fever, Burden estimation, *Plasmodium falciparum*, Fever

## Abstract

**Background:**

The disease burden of *Plasmodium falciparum* malaria illness is generally estimated using one of two distinct approaches: either by transforming *P. falciparum* infection prevalence estimates into incidence estimates using conversion formulae; or through adjustment of counts of recorded *P. falciparum*-positive fever cases from clinics. Whilst both ostensibly seek to evaluate *P. falciparum* disease burden, there is an implicit and problematic difference in the metric being estimated. The first enumerates only symptomatic malaria cases, while the second enumerates all febrile episodes coincident with a *P. falciparum* infection, regardless of the fever’s underlying cause.

**Methods:**

Here, a novel approach was used to triangulate community-based data sources capturing *P. falciparum* infection, fever, and care-seeking to estimate the fraction of *P. falciparum*-positive fevers amongst children under 5 years of age presenting at health facilities that are attributable to *P. falciparum* infection versus other non-malarial causes. A Bayesian hierarchical model was used to assign probabilities of malaria-attributable fever (MAF) and non-malarial febrile illness (NMFI) to children under five from a dataset of 41 surveys from 21 countries in sub-Saharan Africa conducted between 2006 and 2016. Using subsequent treatment-seeking outcomes, the proportion of MAF and NMFI amongst *P. falciparum*-positive febrile children presenting at public clinics was estimated.

**Results:**

Across all surveyed malaria-positive febrile children who sought care at public clinics across 41 country-years in sub-Saharan Africa, *P. falciparum* infection was estimated to be the underlying cause of only 37.7% (31.1–45.4, 95% CrI) of *P. falciparum*-positive fevers, with significant geographical and temporal heterogeneity between surveys.

**Conclusions:**

These findings highlight the complex nature of the *P. falciparum* burden amongst children under 5 years of age and indicate that for many children presenting at health clinics, a positive *P. falciparum* diagnosis and a fever does not necessarily mean *P. falciparum* is the underlying cause of the child’s symptoms, and thus other causes of illness should always be investigated, in addition to prescribing an effective anti-malarial medication. In addition to providing new large-scale estimates of malaria-attributable fever prevalence, the results presented here improve comparability between different methods for calculating *P. falciparum* disease burden, with significant implications for national and global estimation of malaria burden.

**Electronic supplementary material:**

The online version of this article (10.1186/s12936-019-2830-y) contains supplementary material, which is available to authorized users.

## Background

Despite significant gains in reducing malaria prevalence, *Plasmodium falciparum* malaria remains a significant global health burden, causing approximately 216 million cases and 445,000 deaths globally in 2016 [1–3]. For estimation purposes, malaria cases are currently defined as a parasitologically-confirmed *Plasmodium* infection [4], measured in practice from aggregated reports of diagnostic testing amongst fever cases in health clinics reporting to the health management information system (HMIS) in malaria-endemic countries. Current burden estimation methodologies for enumerating total community cases in malaria-endemic countries rely on either: (i) aggregating total numbers of confirmed cases reported to the HMIS, then making adjustments for reporting completeness, unconfirmed cases and treatment-seeking (henceforth “adjusted case-count approach”); or (ii) applying a prevalence-to-incidence conversion [5], whereby cartographic models of *P. falciparum* prevalence [6] are used to determine the number of infections that become symptomatic at a national level (henceforth “cartographic approach”). Evidently, these two methodologies, while both used to estimate national case counts, do not follow the same strict definition of a malaria “case”.

The first step of the adjusted case-count approach enumerates the number of fever cases presenting at clinics that are coincident with a patent *P. falciparum* infection, before adjusting to produce estimates of the total number of community cases. By enumerating *P. falciparum*-positive fevers, no distinction is made between *P. falciparum*-attributable fevers and non-malarial fevers coincident with a diagnostically patent, asymptomatic *P. falciparum* infection, and both are reported as confirmed malaria cases by the clinic to the HMIS.

Conversely, the cartographic approach relies on mechanistic models of the proportion of infected individuals who go on to experience a symptomatic infection [5, 7–9]. By this rationale, the cartographic approach only estimates *P. falciparum*-attributable fevers, and not fevers that are coincident with, but not caused by, a patent *P. falciparum* infection. Burden estimates derived using this methodology will therefore produce estimates of malaria cases that are systematically lower than those produced using the adjusted case-count approach.

The adjusted case-count approach is typically used where observations of community *P. falciparum* prevalence (required to utilize the cartographic approach) are sparse or parasite prevalence is low (e.g. in much of Asia and the Americas) [1, 2, 10]. Conversely, application of the adjusted case-count approach is challenging in many African countries where health system surveillance systems are often incomplete and data may be reported only from some public clinics, often without parasitological diagnosis [3, 10, 11]. The cartographic approach is used more often in these countries, as national household surveys measuring malaria prevalence are common, allowing for robust mapping. However, for many countries with high *P. falciparum* prevalence, there is increasing interest in adopting the adjusted case-count approach into burden estimation frameworks to benefit from the higher cadence of updates and the new digital health information systems technologies in these countries. To examine compatibility between the two approaches, in the most recent World Malaria Report, a number of sub-Saharan African countries reported increasing numbers of malaria cases when estimated via the adjusted case-count approach [3]. When estimated for the same countries via the cartographic approach, however, the same rises were not observed. A better understanding of the difference between the rate of *P. falciparum*-attributable fevers and *P. falciparum*-positive fevers (and by extension, the metrics produced via the adjusted case-count and cartographic approaches) is required to estimate the true burden of malaria.

In this analysis, a model is presented to estimate the proportion of *P. falciparum*-positive fevers that are causally attributable to the *P. falciparum* infection, amongst febrile children under 5 years of age presenting at public health facilities in a number of sub-Saharan African countries. Henceforth, the following terminologies are used: (i) malaria-positive fever, defined as a *P. falciparum*-positive fever, without confirmation of the infection being the underlying cause, (ii) malaria-attributable fever (MAF), defined as a *P. falciparum* infection where infection has been determined to be the fever’s underlying cause, and (iii) non-malarial febrile illness (NMFI), defined as a fever with an underlying non-malarial cause, which may or may not be co-incident with a *P. falciparum* infection.

Here, estimates of malaria-positive and malaria-attributable fevers were collated from a wide variety of sources (with different thresholds for attribution, and differing fever definitions) and the relationship between the proportion of malaria-positive fevers that are causally attributable to malaria in a given community and the corresponding *P. falciparum* parasite prevalence (*Pf*PR) was modelled. This relationship was used to calibrate a Bayesian hierarchical model, which was fit to household survey data to predict whether febrile children under 5 years of age in this dataset had a NMFI, a MAF, or co-symptomatic MAF and NMFI. Individuals’ subsequent treatment-seeking behaviour was then tracked to generate estimates of the proportion of febrile individuals under 5 years of age presenting at health clinics with fevers due to the following underlying causes: (i) MAF, (ii) co-symptomatic MAF and NMFI, (iii) NMFI coincident with a rapid diagnostic test (RDT)-patent asymptomatic *P. falciparum* infection, and (iv) NMFI without an RDT-patent *P. falciparum* infection. These estimates were compared to malaria case counts in children under 5 years of age obtained from Ministry of Health reports from three malaria-endemic countries, to estimate the proportion of malaria-positive fevers with a non-malarial underlying cause. These findings highlight the difference between *P. falciparum*-positive fevers presenting at clinics (considered “cases” under the adjusted case-count approach) and the true burden of symptomatic malaria-attributable infections (the metric estimated under the cartographic approach). From a clinical perspective, both malaria-positive and malaria-attributable fevers should still receive anti-malarial medication, but the results presented in this analysis indicate the proportion of fevers coincident with *P. falciparum* infections that would not be resolved by anti-malarial medication alone. Many of these fevers are likely to be caused by self-limiting infections and not require further treatment, but nevertheless these findings highlight the importance of clinicians checking for signs of other infections even when *P. falciparum* infection is diagnosed.

## Methods

### Modelling the relationship between malaria-attributable fever and malaria prevalence

Four different primary datasets were assembled and used to measure the relationship between MAF and *P. falciparum* prevalence in children under 5 years of age (*Pf*PR_0–5_). The four datasets were chosen to encompass the variety of definitions of fever (i.e. measured or recalled), and differing methods for defining MAF. The datasets fell broadly into two categories: defining MAF using a pyrogenic parasite density threshold for malaria-infected individuals, above which malaria-positive fevers are defined as MAF (one dataset); and datasets which record paired observations of malaria positivity and fever (either measured fever at the point of diagnosis, or recalled recent fever history) allowing for empirical estimation of MAF (three datasets). Full details of the four datasets are given in Additional files 1, 2, 3.

The first dataset, comprised of observations of *P. falciparum* infections above and below a pyrogenic parasite density threshold, was derived from active case detection studies. In this dataset, the proportion of malaria-infected individuals under and over the pyrogenic threshold was used in conjunction with the *Pf*PR at the study site in the modelling approach described below. Details of the parasite density threshold dataset can be found in Additional file 1.

The remaining three datasets consisted of paired records of fever and malaria positivity. From these, the proportion of malaria-positive fever cases where the malaria infection was the underlying cause of the fever (MAF) was derived empirically by accounting for the rate of malaria-negative fever in the population. This empirical calculation assumes that the rate of fever caused by NMFI is the same in both the *P. falciparum*-positive and *P. falciparum*-negative populations, and as such the “excess” fevers within the febrile *P. falciparum*-positive individuals not explained by the background NMFI rate are considered to be fevers that are causally attributable to the *P. falciparum* infection. Full details of each of these three datasets and the formula for empirical calculation of MAF are given in Additional files 1, 2, 3, 4. Briefly, all three datasets were composed of observations from study sites where *Pf*PR_0–5_ was measured, and paired observations of malaria positivity and fever were recorded in children under five. The first dataset consisted of household survey data from the Demographic and Health (DHS) Program (156,670 children; Additional file 2). The second dataset consisted of observations extracted via two systematic searches of literature (123,649 individuals in 94 study sites from 33 publications; Additional file 3). The third database was sourced from the *Programme for Resistance, Immunology, Surveillance and Modeling of Malaria in Uganda* (PRISM) study [12] (3 study sites, 2804 individuals; Additional file 3). Combined, the literature review and PRISM datasets yielded observations from a total of 112 study sites, but only 27 were used for modelling. Exclusion reasons included a focus on individuals 5 years of age or above (n = 82 excluded), or malaria diagnosis using only RDT or PCR (n = 3). As the majority of observations were obtained via microscopy, all other diagnostic methods were excluded to retain consistency within the literature review and PRISM datasets. Full details of the literature review data, including observations amongst adults and children over 5 years of age not included in the final analysis, can be found in Additional file 3.

Using the four datasets described above, the relationship between MAF in children under 5 years of age and *Pf*PR_0–5_ was learned in a two-step Bayesian model. In the first step, the DHS Program data was used to learn the underlying shape of the relationship between MAF (as a proportion of malaria-positive fevers, amongst children under 5 years of age) and *Pf*PR_0–5_. Next, the remaining three datasets (the parasite density dataset, literature review dataset, and the PRISM dataset) were used in a second fitting step to re-scale the relationship learned in the first step. Full details of the model fitting methodology are given in Additional file 4. Use of these three datasets in a second fitting step was essential to adjust for a “treatment effect” introduced by the DHS Program data. In the DHS Program surveys, RDT diagnosis is conducted at the time of interview, but fever positivity is measured via a 2-week recall. As such, a malaria-positive febrile child at a previous point during the 2 weeks preceding the interview may have sought and received anti-malarial treatment, and their blood antigen concentration may have reduced sufficiently to appear RDT-negative by the time of interview; this is the “treatment effect”.

Two additional factors which may impact the relationship between MAF and *Pf*PR_0–5_ were also investigated: (i) the effect of residual exposure-dependent immunity, measured using recent declines in *Pf*PR_0–5_, and (ii) the effect of background treatment rate. Data sources for each of these factors can be found in Additional file 4.

### MAF–NMFI model and generation of clinic-level estimates

A Bayesian hierarchical model was used to assign the probability of each febrile child in a household survey dataset having experienced one of the four following fever types during the 2 weeks preceding the household survey: (i) MAF, (ii) co-symptomatic MAF and NMFI, (iii) NMFI coincident with a rapid diagnostic test (RDT)-patent asymptomatic *P. falciparum* infection, and (iv) NMFI without an RDT-patent *P. falciparum* infection. Each child’s treatment-seeking behaviour was tracked in order to establish the proportion of malaria-attributable and non-malarial fevers presenting at health facilities. To inform the model, the DHS Program household survey dataset described in the previous sub-section was utilized (Additional file 2). From the DHS Program surveys, the following information was used: (i) RDT-derived parasitaemia, (ii) and 2-week fever history, as recalled by the child’s caregiver, (iii) a geo-referenced location (for matching with remotely sensed explanatory variables), (iv) information about treatment-seeking behaviour at formal public health clinics that would likely report to the HMIS (locations such as shops, pharmacies, markets, drug peddlers, friends and relatives, or traditional healers, and formal private health clinics were not included in this analysis), and (v) information about the child’s age, household wealth, maternal education, and child usage of insecticide-treated bed nets the night preceding the survey. A total of 156,670 observations were collated from the 41 surveys.

In the Bayesian hierarchical model, three model-derived spatial covariates were used as predictors of MAF or NMFI. These three predictor variables were: (i) *Pf*PR_0–5_, (ii) suitability for fever without a malaria infection in children under 5 years of age, and (iii) caregivers’ propensity to seek treatment for fever of any cause for children less than 5 years of age. All three covariates were available for each year of the study period at a 5 × 5 km spatial resolution across areas of stable *P. falciparum* transmission in sub-Saharan Africa. The value of each of the three covariates was extracted for each child based on their GPS location and the time of survey. Full details of each covariate are given in Additional file 5.

The Bayesian hierarchical model utilized in this analysis is an adaptation of a previously presented model for estimating the prevalence of MAF and NMFI across sub-Saharan Africa from 2006 to 2014 [13]. Here, the Bayesian hierarchical model was recalibrated using the fitted relationship between MAF and *Pf*PR described in the previous subsection. By using this recalibration (and not fitting the model on DHS Program data alone, as had been done previously [13]), the model is able to account for biases intrinsic in the DHS Program data, such as the up-to-14 day lapse between fever recall and malaria testing, and to account for different methods of defining MAF (either through empirical calculation, or through defining a parasite density threshold above which a fever is caused by malaria). This improved model also incorporated additional individual-level data on socioeconomic status and treatment-seeking behaviour in the 2 weeks preceding the survey. An estimation strategy was used where Bayesian posterior samples of predicted fever type (i.e. MAF or NMFI) for each febrile individual within the household survey were compiled using the design weights of the original survey to derive nationally-representative estimates of the proportion of malaria-positive fevers presenting at clinics that were causally attributable to malaria. Finally, the model was further augmented to adjust for the probability of a child having been malaria-positive and febrile in the previous 2 weeks, having received treatment, and reverting to RDT-negative by the time of interview. The adjustment procedure calculated the probability of reversion depending on both the type of antimalarial medication received and the type of RDT used at the point of interview. Full model specification can be found in Additional file 6.

For country-years where a contemporaneous Ministry of Health report was publicly available, the modelled proportion of malaria-positive fevers that were/were not causally attributable to malaria was applied to the reported number of malaria-positive fevers amongst children under five from each report. Ministry of Health reports were identified via Google search for all countries with a survey, and included if annual confirmed malaria cases were reported for children under five.

## Results

### Relationship between malaria-attributable fever and *P. falciparum* prevalence

**Fig. 1:**
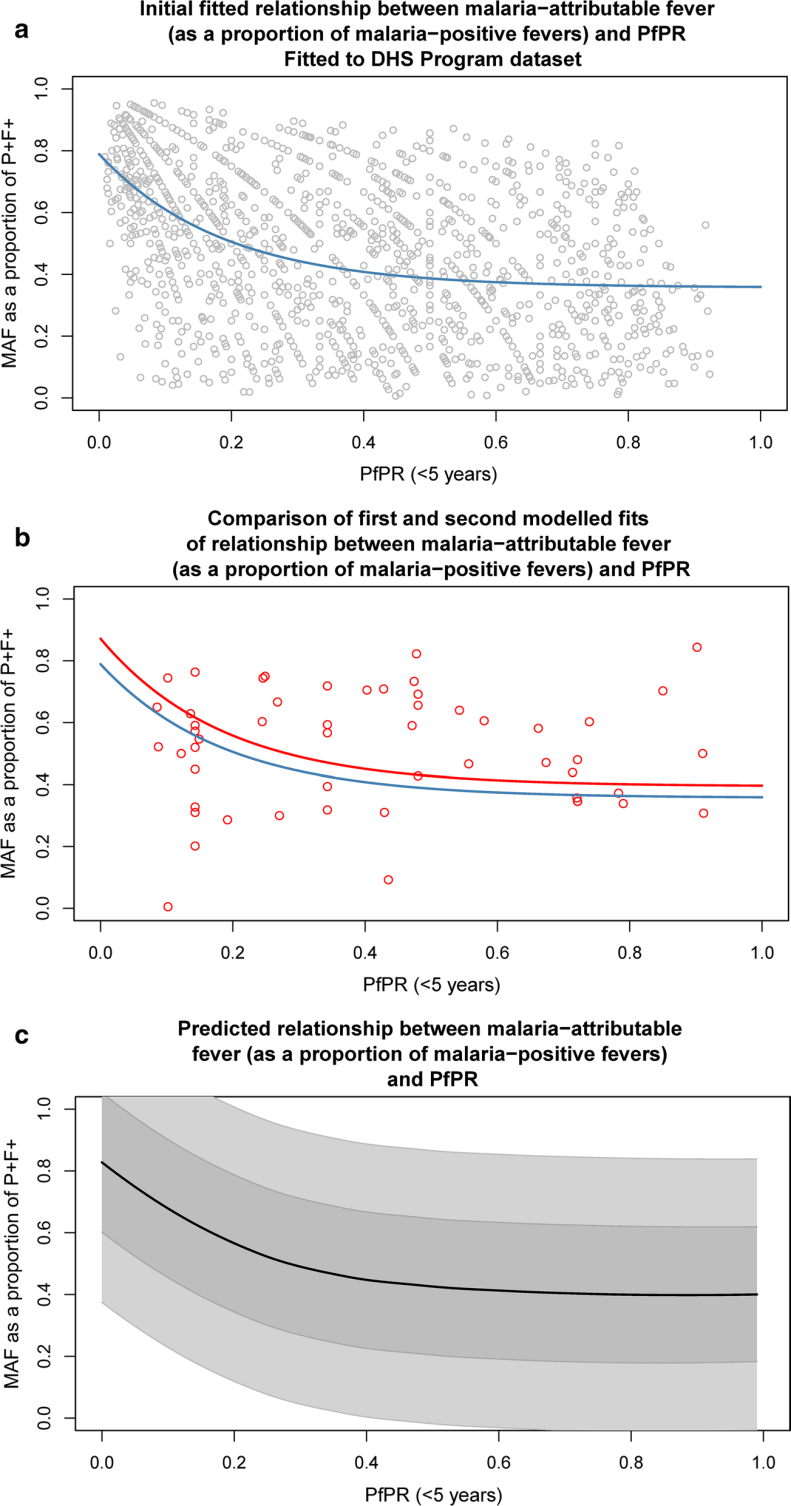
Fitted relationship between MAF and *Pf*PR_0–5_. **a** Initial modelled median relationship (blue line) between MAF (as a proportion of malaria-positive fevers) in children under 5 years of age, and *Pf*PR_0–5_, fit only to empirical observations of MAF from the DHS Program household survey dataset, with survey data-points (grey points) overlain; **b** original modelled median relationship as described in the previous plot (blue line), and final rescaled model median fit (red line), after optimizing scaling parameters using the remaining three literature-derived datasets. Observation points from the three literature-derived datasets are overlain (red points); and **c** final fitted relationship between MAF (as a proportion of malaria-positive fevers) in children under 5 years of age, and *Pf*PR_0–5_. The black line represents the median, with decreasing grey shading representing the 68% and 95% credible intervals, respectively

The proportion of malaria-positive fevers that are causally attributable to malaria was estimated to decline exponentially from 0.82 at *Pf*PR_0–5_ ~ 0, until *Pf*PR_0–5_ > 0.4 after which the proportion declines much more slowly. The coefficients for the fitted model are given in Table 1, and the final fitted model (with 68% and 95% credible intervals) is shown in Fig. 1.

**Table 1:** Final model coefficients

Coefficient	Value
*β*_*final*_	0.477
*λ*	5.354
*α*	0.827
σ	0.215
*β*_*Initial*_	0.432

*β*_*final*_ represents the value of the scaling parameter, *β*, after the second model-fitting (to the literature review data) and *β*_*Initial*_ represents the value of *β* after the first model fitting (to the DHS Program data), which is not used in the final model fit. The parameter *λ* controls the rate of decline of the proportion of MAF within malaria-positive fevers with increasing *Pf*PR_0–5_. The parameter *α* controls the minimum proportion of malaria-positive fevers in a population that can be due to malaria. The parameter σ is the standard deviation/observational noise parameter. Full details of model fitting and parameter optimisation can be found in the Additional file 6

**Fig. 2:**
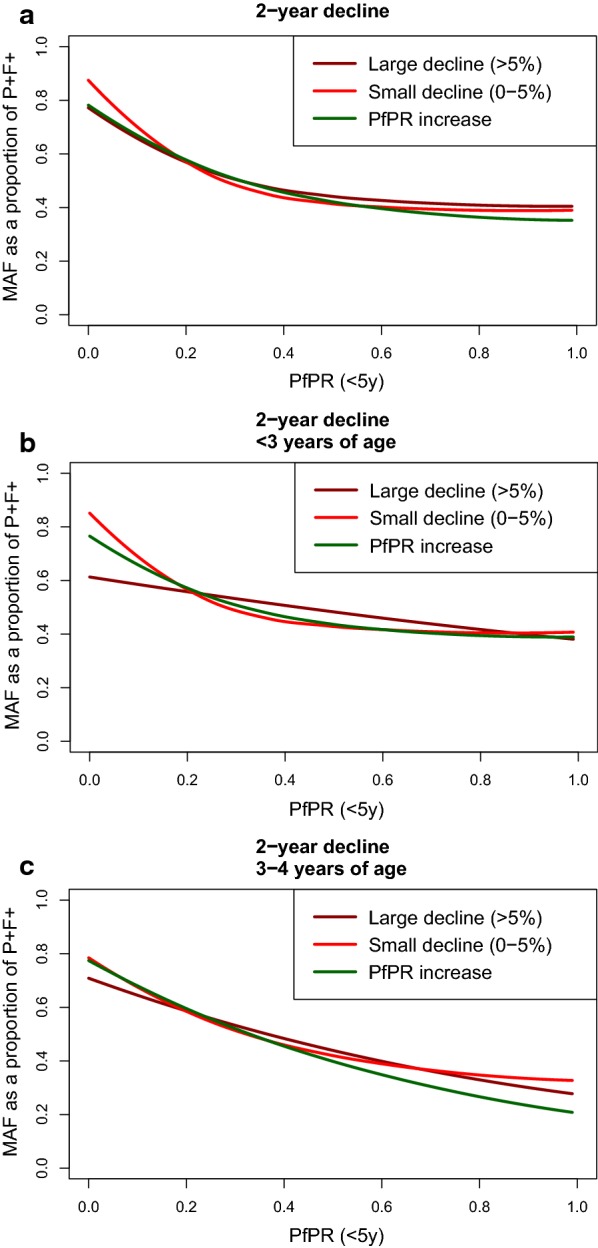
Fitted relationship between MAF and *Pf*PR_0–5_ when stratified by the magnitude of *Pf*PR_0–5_ decline in the 2 years preceding data collection. The top panel (**a**) shows the fitted relationship for all children under 5 years of age, with children located in areas where *Pf*PR_0–5_ declined by more than 5% in the 2 years preceding the survey in dark red, children located in areas where *Pf*PR_0–5_ declined by between 0 and 5% in red, and children located in areas where *Pf*PR_0–5_ increased in green. In the central panel (**b**) and bottom panel (**c**) the three decline categories are indicated by the same colours as **a**; **b** shows the relationship only amongst children aged 2 years or younger, and **c** shows the relationship only amongst children aged 3 or 4 years. Declines in *Pf*PR_0–5_ were extracted from annual *Pf*PR predictions produced by Bhatt et al. [6]

Recent declines in *Pf*PR_0–5_ (Fig. 2) caused limited effects, with non-significant differences between the relationships when fitted to DHS Program data points in locations that had experienced large declines in *Pf*PR_0–5_ (> 5% reduction in *Pf*PR_0–5_ in the 2 years preceding the survey), compared to lesser declines (< 5% reduction) or *Pf*PR_0–5_ increases. Differences in the fitted relationship by age were observable with older children (3 or 4 years of age; Fig. 2c) experiencing continual declines in the proportion of malaria-positive fevers that are causally attributable to malaria with increasing *Pf*PR_0–5_, whereas children 2 years of age or younger (Fig. 2b) experienced rapid exponential declines until approximately *Pf*PR_0–5_ > 0.4. Older children showed a lower proportion of malaria-positive fevers causally attributable to malaria infection at higher *Pf*PR_0–5_.

**Fig. 3:**
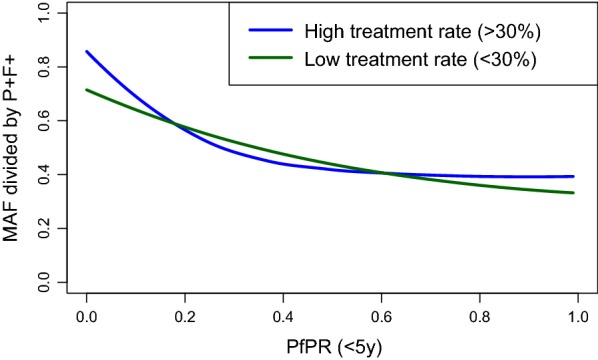
Fitted relationship between MAF and *Pf*PR_0–5_ when segregated by treatment-seeking rate within DHS Program clusters. The blue line represents the fitted relationship for clusters where the treatment-seeking rate for febrile children was ≥ 30%, and the green line for clusters where the treatment-seeking rate was < 30%

Background treatment-seeking rate for fever (used as a proxy for underlying treatment rate for malaria infections) was not found to have a significant impact on the relationship between malaria-attributable fever (as a proportion of malaria-positive fevers) and *Pf*PR_0–5_. Figure 3 shows the relationship between malaria-attributable fever (as a proportion of malaria-positive fevers) and *Pf*PR_0–5_ for DHS Program clusters where the treatment-seeking rate for febrile illnesses within the cluster was over and under 30% (a threshold chosen based on the 50% quantile of the proportion of febrile children sought treatment across all the household survey clusters, grouping the clusters into equally sized higher- and lower-treatment groups), showing non-significant differences between the two subsets.

### Ratio of MAF and NMFI amongst febrile children presenting to public health clinics

The final fitted relationship between malaria-attributable fever (as a proportion of malaria-positive fevers) and *Pf*PR_0–5_ was implemented in the Bayesian hierarchical model to attribute fevers amongst the surveyed individuals to either malaria or non-malarial causes. For each survey, the treatment-seeking rate of MAF and NMFI cases (which can be RDT-positive or RDT-negative) was collated to produce an estimate of the proportion of each fever type presenting at public health clinics in that country-year.

**Fig. 4:**
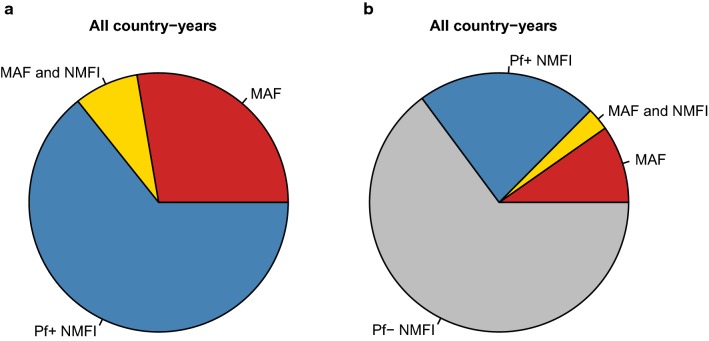
Estimates of proportion of fevers presenting to formal public health clinics that are causally due to malaria versus non-malarial febrile illness. **a** Estimates of proportion of fevers presenting to public health clinics that are causally due to MAF (red sections), NMFI accompanied by an asymptomatic *P. falciparum* malaria infection (blue sections) and co-symptomatic MAF and NMFI (yellow sections); and **b** as **a**, also including NMFI not accompanied by a *P. falciparum* malaria infection (grey sections); amongst febrile children under 5 years of age presenting to formal public health clinics amongst children from household surveys in 41 country-years in sub-Saharan Africa, 2006–2016

Averaging across all surveys, Fig. 4 illustrates the proportion of fevers estimated to fall into each of the four categories: (i) MAF only, (ii) fever causally due to co-symptomatic MAF and NMFI, (iii) NMFI coincident with an RDT-patent asymptomatic malaria infection, and (iv) NMFI without an RDT-patent malaria infection. Proportions of each fever type for each of the 41 surveys can be found in Additional file 8. On average, 37.7% (31.1–45.4, 95% CrI) of malaria-positive fevers presenting at clinics were causally attributable to malaria, ranging from 5.3% (1.8–12.4, 95% CrI) in Tanzania in 2007–2008, to 81.5% (77.7–84.5, 95% CrI) in Mali in 2012–2013. These estimates suggest that the burden of malaria, if estimated using the adjusted case-count approach, may significantly overestimate the burden of symptomatic *P. falciparum* malaria if all malaria-positive fevers were counted as malaria cases.

Amongst the surveyed country-years, six countries were surveyed three or more times between 2006 and 2016 (Angola, Liberia, Madagascar, Senegal, Tanzania, Uganda). The proportion of malaria-positive fevers that were predicted to be causally due to malaria rose in Angola, Madagascar, Senegal (with the exception of 2010–2011), and Tanzania between the first and last surveys. This was consistent with a decrease in *Pf*PR_0–5_ over the survey time period in those countries [6] and reflects the predicted relationship between MAF and *Pf*PR_0–5_ shown in Fig. 1. Uganda showed a rise in the proportion of malaria-positive fevers that were predicted to be causally due to malaria between the first and second survey, but this proportion dropped again slightly between the second and third survey. Liberia showed a relatively constant proportion of malaria-positive fevers predicted to be causally due to malaria across all three surveys (Additional files 7, 8). Fourteen of the 41 country-years had a higher proportion of malaria-positive fevers presenting at clinics than malaria-negative fevers, with, on average for all countries, 35.1% of fevers presenting at public clinics being accompanied by a malaria infection (Additional files 7, 8). Amongst children with NMFI (i.e. fevers where malaria was not the underlying cause of the fever) across all surveyed country-years, 26.8% (25.5–27.9, 95% CrI) were accompanied by an RDT-patent malaria infection. This fraction varied significantly between country years, from 1.0% (0.4–1.4%, 95% CrI) in Senegal in 2015, to 66.4% (65.7–66.9, 95% CrI) in Burkina Faso in 2014 (Additional files 7, 8).

### Comparison with confirmed cases reported in malaria-endemic countries

**Fig. 5:**
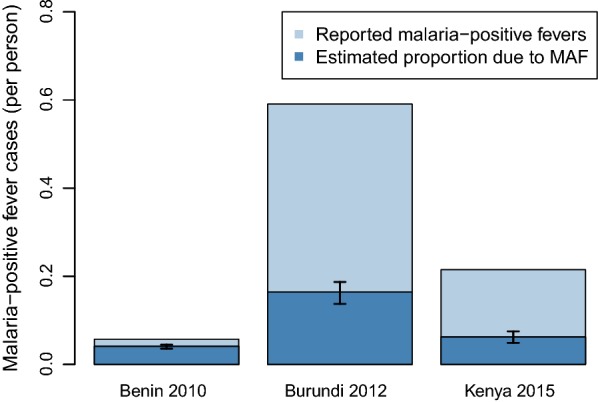
Proportion of reported malaria cases in three countries that were truly symptomatic malaria infections versus asymptomatic malaria infections coincident with non-malarial febrile illnesses. The incidence of malaria-positive fevers per child under five reported to HMIS in Benin 2010 (left bar), Burundi 2012 (middle bar) and Kenya 2015 (right bar) amongst children under 5 years of age in light blue bars, with the total number of malaria-attributable fevers overlain in dark blue bars. The upper and lower 95% credible intervals on the estimate of number of malaria-attributable fevers are given by black error bars. Malaria-positive fevers not causally attributable to fever were predicted to be caused by non-malarial febrile illnesses

Confirmed malaria cases (i.e. fever cases presenting to clinics with a positive malaria diagnosis) amongst children under 5 years of age were available for three country-years with near-contemporaneous household surveys (Benin 2010 [14], Burundi 2012 [15] and Kenya 2015 [16]). In 2010, Benin reported 90,736 cases amongst children under 5 years of age; in 2012, Burundi reported 1,138,088 cases amongst children under 5 years of age; and in 2015, Kenya reported 1,569,892 cases amongst children under 5 years of age. Figure 5 displays the incidence of malaria-positive fevers presenting at clinics in the three countries, separated into malaria-attributable fevers, and malaria-positive fevers not caused by the malaria infection. It is estimated that 28.2% (25,572 malaria-positive fevers, 19,487–33,939, 95% CrI) of malaria-positive fevers in Benin in 2010, 72.2% (821,270 malaria-positive fevers, 777,791–873,594, 95% CrI) malaria-positive fevers in Burundi in 2012, and 71.1% (1,116,756 malaria-positive fevers, 1,023,064–1,213,370, 95% CrI) of malaria-positive fevers in Kenya in 2015 amongst children under 5 years of age reported to the HMIS in each country were causally attributable to non-malarial causes. As such, fever would not be resolved through effective anti-malarial medication and would require additional treatment for the underlying non-malarial febrile illness to be resolved.

## Discussion

### Interpretation and implications

The analysis presented here indicates that a significant proportion of confirmed *P. falciparum* malaria cases reported by the HMIS in malaria-endemic countries are likely to be non-malarial fevers coincident with an asymptomatic *P. falciparum* infection. Across all surveyed country-years, the majority of febrile children for whom treatment was sought did not have a malaria-positive fever, and amongst the children who did have a malaria-positive fever, the malaria infection was not the underlying cause for nearly two-thirds of these fevers. It is important to note that in this analysis, a symptomatic malaria infection is defined solely by febrile illness, and as such, other symptoms of chronic malaria infections (such as anaemia) are not considered.

In this analysis, prevalence of malaria-attributable fever amongst malaria-positive children who sought treatment during the 2 weeks preceding each survey was used to generate an incidence of malaria-attributable and malaria-positive fever at clinics, applicable only to the 2 weeks preceding each survey. Although conventional burden estimates quantify the number of malaria cases over the course of a year, these findings signify that the adjusted case-count approach for burden estimation may significantly overestimate the clinical malaria burden by classing all fevers with a patent malaria infection as a “malaria case”. The reasons for differential proportions of MAF and NMFI amongst malaria-positive fevers between countries are complex and depend on both the national prevalence of both *P. falciparum* and NMFI. Many of the surveys from countries with the greatest predicted overestimates for cases using the adjusted case-count approach were completed in the first half of the study decade, coinciding with higher malaria burden (and therefore higher levels of exposure-related immunity [6]) and worse sanitation and housing conditions relative to later years [17]. These countries also typically experience higher rates of non-malarial febrile illness. The comparison of MAF and NMFI fractions of clinic-based confirmed cases to contemporaneous malaria cases reported to three Ministries of Health in sub-Saharan Africa (Fig. 5) shows the potential for malaria-positive fevers that are not causally attributable to malaria to be systematically reported as “malaria cases”, which may lead to significant overestimations of the clinical burden of malaria.

### Methodological discussion and limitations

Many of the 21 countries included in this study were surveyed multiple times between 2006 and 2016, with three surveys in Angola, Liberia, Madagascar, Tanzania and Uganda, and four surveys in Senegal. In most of these countries, a larger fraction of malaria-positive fevers were causally attributable to malaria in later surveys compared to earlier surveys within the same country (with the exception of 2016 surveys in Tanzania and Uganda). These findings may be explained by a loss of exposure-related immunity amongst children under 5 years of age as malaria prevalence decreases (all six countries showed decline in *Pf*PR_0–5_ over the study period) [6, 18] resulting in a higher fraction of symptomatic illness amongst infected children (although this pattern was not observed in some other countries where *Pf*PR_0–5_ decreased over the study period). This increase in MAF in the six countries could also be explained by a decrease in NMFI prevalence relative to malaria prevalence over the time period, but this was not observed in the current study [13]; indeed in most countries, a higher fraction of NMFI compared to MAF was observed amongst febrile children presenting at public health clinics in more recent country-years. A possible limitation of this repetitive surveying through time and space is that the results may not be easily applied to other countries with similar levels of malaria transmission intensity; indeed, this study shows that transmission intensity is not the only factor which affects the proportion of malaria-positive fevers that are attributable to malaria, and that non-malarial fever prevalence and household characteristics also play a role. The model included a survey-level random effect to account for biases introduced by the blocking of surveys through time and space.

Averaging over the 41 surveyed country-years, approximately two-thirds of malaria-positive fevers amongst children under 5 years of age would not be resolved with effective anti-malarial treatment. If clinicians do not investigate other causes of fever given a positive RDT, non-malarial fevers may be systematically missed and remain untreated, potentially leading to missed opportunities to reduce the burden of other important non-malarial febrile illnesses. It is, however, also important to consider that many of the underlying causes of these non-malarial fevers may not warrant treatment, for example if the underlying cause is a self-limiting illness. As such, although this analysis suggests that clinicians should be aware that co-infections with *P. falciparum* and other illnesses are common, the results presented here do not directly indicate any requirement for upscaling of antimicrobial medications, especially in areas where bacterial infections are less common. Evidence from literature suggests that clinicians show good adherence to integrated management of childhood illness (IMCI) guidelines when prescribing ACT after a positive RDT [19, 20], but information is sparse on clinicians’ propensity to investigate alternative causes of illness after either negative or positive RDT outcomes. Evidence suggests that in malaria-endemic areas, a shift from malaria-centric test-and-treat methods towards a holistic IMCI approach in clinics would both improve health outcomes and reduce unnecessary antibiotic use [21]. Studies have shown that clinicians are more likely to prescribe antibiotics to patients with a negative RDT than malaria-positive patients in a study in Zanzibar [20], and that after IMCI training clinicians do record diagnoses of malaria co-infections with NMFI [22]. Diagnosis of common NMFIs is hampered by a lack of routine diagnostics that are cost-effective at the clinic level, and further challenged by the diverse and heterogenous fever aetiology across the continent [23]. This problem may also be exacerbated by health systems that often require a single cause for febrile illness to be reported, which may lead to an over-reporting of malaria in areas of high prevalence, and an artificial boost of NMFIs in areas where malaria prevalence is lower. The applicability of these results to age groups older than 5 years of age is ambiguous, owing to the lack of information on treatment-seeking rates for older ages.

In this analysis, a number of additional factors hypothesized to affect the relationship between MAF and *Pf*PR were investigated. No effects of recent *Pf*PR declines or differential underlying treatment rates were observed on the relationship between MAF and *Pf*PR (Figs. 2, 3). It is unclear whether these effects were not observed because they do not affect the relationship, were due to insensitivity of the modelling framework, or were due to the calibration dataset being ill-adapted for measuring the effect of recent declines in *Pf*PR. Multiple sets of longitudinal cohort studies at differing levels of baseline *P. falciparum* exposure would be better-suited to measure the effect of recent *Pf*PR declines on the relationship between MAF and *Pf*PR as they capture the changing immunity profile of individuals over time within the same cohort. Mechanistic models suggest a loss of immunity following up-scaling of malaria interventions, and a shift towards increased levels of severe malaria-attributable fever in older children following declines in *P. falciparum* exposure [24]. Any lag between reduction in *Pf*PR and this loss of exposure-related immunity would imply that the profile of the relationship between MAF and *Pf*PR might temporarily shift until exposure-related immunity reaches a new equilibrium.

The effect that high or low levels of community anti-malarial treatment may have on the relationship between MAF and *Pf*PR is unclear. High levels of prompt and effective treatment may reduce the exposure-related immunity in a community, altering the relationship between MAF and *Pf*PR. Here, the treatment-seeking rate for fever was used as a proxy for the level of prompt and effective treatment, and no effect of treatment was found on the relationship between MAF and *Pf*PR. Case–control studies with prompt treatment of fevers may increase the sensitivity of the approach used here.

The household survey datasets used in this analysis report RDT result from the time of interview, but caregiver recall of fever episodes within the past 2 weeks (rather than fever measurement at the time of interview). In addition to biases in recall of recent fever [25, 26] the possibility exists that an individual with a patent malaria-attributable fever may seek and receive effective anti-malarial treatment, and return to RDT-negative by the time of interview, despite having experienced a malaria infection in the 2 weeks preceding the survey. If left unadjusted, an overestimation of the proportion of NMFI amongst the individuals who sought and received treatment would be expected. The modelling study presented here is the first of its kind to adjust for the possibility of reversion from RDT-positive to RDT-negative within the 2 weeks preceding the survey, as determined using a previously presented model [27]. Datasets with RDT outcome at the point of care would increase the sensitivity of this approach, although diagnostic testing for all fever cases presenting at clinics is not universal; amongst febrile individuals who sought care at a public clinic in the household survey dataset only 44.3% had blood drawn at the point of care (used in this study as a proxy for parasite-based malaria diagnosis). The possibility exists that, under this approach, an individual may have been treated for a malaria-positive fever over 14 days prior to the interview (and, therefore, not report any fever, or treatment-seeking) yet still present a positive RDT at the time of interview, due to the persistent positivity of RDTs after treatment [27]. This potential limitation is not addressed in the current study, although the effect is estimated to be small, given the low treatment-seeking rate for malaria-positive fevers, and the lower-still proportion who received an effective anti-malarial [3].

Fever caused by *Plasmodium vivax* malaria is also, by definition, grouped within NMFI in this analysis, confounding the definition of NMFI. *P. vivax* is not prevalent in Africa (in part due to widespread Duffy negativity [28]), except in Mauritania and East Africa [29, 30]. Due to location of the surveyed country-years represented here (which mostly fall outside the zones of stable *P. vivax* transmission [31]), the effect of *P. vivax*-attributable fevers in the country-years represented in this analysis is presumed to be limited. Caution should, however, be taken when extending the approach presented here to areas of higher *P. vivax* burden.

The results presented here suggest a high prevalence of co-infections between malaria and NMFI amongst febrile children presenting at clinics. Here, the effects of co-morbidity (or modulating effects of co-infection on febrile illness) are not accounted for, owing to the lack of diagnosis for NMFI. Future studies may aim to investigate the effects of co-morbidity if differential diagnosis of NMFI is available, or to include spatial distributions of known NMFI-causing pathogens, which were precluded from the current analysis given the lack of availability of cartographic NMFI estimates at the same scale as the *Pf*PR model inputs.

The Ministry of Health reports used to generate the number of paediatric malaria-positive fevers that would and would not be resolved by anti-malarial treatment alone in three sub-Saharan African countries (Fig. 5) are subject to biases not adjusted for in this study. Although the reports document the total number of cases reported from health facilities, not all health facilities report to the HMIS. Biases in which facilities report to the HMIS may mean the estimates presented here are not representative on a national scale. If certain facilities (e.g. those in urban areas, typically associated with a lower *P. falciparum* burden [32], or those receiving more severe malaria cases, such as hospitals) are not equally likely to report to the HMIS than rural facilities, the estimates may not be nationally representative. The estimates for the proportion of the annual cases that are estimated to have been malaria-attributable may also be biased by the short data collection period for each survey. Each survey is collected over a period of usually 2–4 months, with fever and care-seeking information for each child only being valid for the 2 weeks preceding each child’s interview. For this reason, the estimates presented here for the proportion of children with MAF seeking care is only valid for the 2 weeks preceding each survey. Because treatment-seeking rates for MAF may vary over the course of a year, the 2-week period of the survey may not necessarily be representative of the treatment-seeking rate across the year. A more accurate comparison would be to calculate a country-specific incidence rate for each of the three countries to compare with the actual reported malaria cases from the three case study countries, but this was not possible with the datasets and modelling methods used in this analysis.

## Conclusions

These findings indicate a need for standardizing approaches for estimating malaria burden. Epidemiologists and public health professionals comparing burden estimates made using the adjusted case-count or cartographic approaches should be aware that these approaches estimate fundamentally different metrics. Refinement of burden estimation approaches to generate estimates of both malaria-attributable fever and malaria-positive fever would improve cross-comparability and allow assessment of the quantity of non-malarial febrile illness amongst malaria-positive fevers. The findings presented here are not intended to suggest that malaria-infected individuals should not be treated with effective anti-malarial medication; but rather the intention of this analysis is to increase understanding of the complex nature of the malaria burden, and to highlight the vast burden of non-malarial paediatric fever.

## Additional files

**Additional file 1.** Studies and data used for parasite density threshold dataset. For each study, the study *P. falciparum* prevalence in children under 5 years of age (*Pf*PR_0–5_), parasite density threshold used, upper and lower age bounds, and the number or proportion of malaria-positive febrile individuals over and under the parasite density threshold is given.

**Additional file 2.** Household survey data utilised for (i) modelling relationship between MAF and *Pf*PR, and (ii) modelling proportion of MAF and NMFI cases seeking treatment in clinics. P+/− refers to the number of children *P. falciparum* positive/negative at time of interview. F+/− refers to the number of children for whom fever was/was not recalled in the 2 weeks preceding the survey.

**Additional file 3.** Literature review and PRISM datasets utilised for modelling relationship between MAF and PfPR. For each study, the fever and parasite positivity status for each individual was extracted. Not all datapoints listed here were used in the final model; analysis was restricted to children under 5 years of age. *Search methodology for datapoint, as documented in “Methods”. **PRISM Data can be downloaded from the ClinEpiDb website: https://clinepidb.org/.

**Additional file 4.** Datasets and modelling approach for estimation of the relationship between malaria-attributable fever and *Plasmodium falciparum* prevalence in children under 5 years of age.

**Additional file 5.** Details of covariates used in MAF–NMFI modelling approach.

**Additional file 6.** Model for individual-level attribution of MAF and NMFI to febrile children within the household survey dataset.

**Additional file 7: Figure S1.** (a) Estimates of proportion of fevers amongst malaria-positive presenting to public health clinics that are causally due to MAF (red sections), NMFI accompanied by an asymptomatic *P. falciparum* malaria infection (blue sections) and co-symptomatic MAF and NMFI (yellow sections); and (b) as (a), also including NMFI not accompanied by a *P. falciparum* malaria infection (grey sections); amongst febrile children under 5 years of age presenting to public health clinics amongst children from household surveys in 41 country-years in sub-Saharan Africa, 2006–2016.

**Additional file 8.** Proportion of each type of fever presenting at public health clinics in each survey, with associated 95% credible intervals. MAF = malaria-attributable fever; NMFI = non-malarial febrile illness; Pf+/Pf− = *P. falciparum* RDT positive or negative (at time of seeking treatment). “MAF and NMFI” indicates febrile children with co-symptomatic MAF and NMFI.

## Data Availability

The datasets generated and/or analysed during the current study are available in the DHS Program repository, https://dhsprogram.com/.

## Abbreviations

HMIS: health management information system
*Pf*PR: *Plasmodium falciparum* parasite prevalence
NMFI: non-malarial febrile illness
MAF: malaria-attributable fever
RDT: rapid diagnostic test
PRISM: Programme for Resistance, Immunology, Surveillance and Modelling of Malaria in Uganda
PCR: polymerase chain reaction
DHS: Demographic and Health Surveys
