# Supplementary material for: The contribution of non-malarial febrile illness co-infections to Plasmodium falciparum case counts in health facilities in sub-Saharan Africa
Source: Malar J. 2019 Jun 11;18:195. doi: 10.1186/s12936-019-2830-y (PMC6560910; doi:10.1186/s12936-019-2830-y)
Supplement: Supplementary file 1 — Additional file 1. Studies and data used for parasite density threshold dataset. For each study, the study P. falciparum prevalence in children under 5 years of age (PfPR0–5), parasite density threshold used, upper and lower age bounds, and the number or proportion of malaria-positive febrile individuals over and under the parasite density threshold is given. [file 12936_2019_2830_MOESM1_ESM.pdf]

Additional File 1. Studies and data used for parasite density threshold dataset. For each study, the study *P. falciparum* prevalence in children under 5 years of age (*Pf* PR_{0-5}), parasite density threshold used, upper and lower age bounds, and the number or proportion of malaria-positive febrile individuals over and under the parasite density threshold is given.

ID	Reference	Study	Site	Country	Publication Year	Year(s) studied	*Pf* PR_{0-5}	Parasite density threshold	AgeLow	AgeUp	*Pf* Pos under threshold	*Pf* Pos over threshold	Numeric or Proportion	Proportion over threshold
1	1	Bloland	Asembo Bay	Kenya	1999	1992	0.7049	1500	0	0.4	222	320	N	0.59
2	1	Bloland	Asembo Bay	Kenya	1999	1992	0.7049	6000	0.5	0.9	221	307	N	0.58
3	1	Bloland	Asembo Bay	Kenya	1999	1992	0.7049	7000	1	2	118	179	N	0.60
4	1	Bloland	Asembo Bay	Kenya	1999	1992	0.7049	7000	2	3	96	57	N	0.37
5	1	Bloland	Asembo Bay	Kenya	1999	1992	0.7049	3500	3	4	191	98	N	0.34
6	2	Lusingu	Mgome	Tanzania	2004	2001	0.8120	5000	0	0.9	9.33	16.57	N	0.64
7	2	Lusingu	Mgome	Tanzania	2004	2001	0.8120	5000	1	1	7.25	17.13	N	0.70
8	2	Lusingu	Mgome	Tanzania	2004	2001	0.8120	5000	2	2	3.15	17.00	N	0.84
9	2	Lusingu	Mgome	Tanzania	2004	2001	0.8120	5000	3	3	1.95	1.95	N	0.50
10	2	Lusingu	Mgome	Tanzania	2004	2001	0.8120	5000	4	4	5.40	2.40	N	0.31
11	2	Lusingu	Ubiri	Tanzania	2004	2001	0.2409	1000	2	2	1.52	3.03	N	0.67
12	2	Lusingu	Ubiri	Tanzania	2004	2001	0.2409	1000	4	4	2.73	1.17	N	0.30
13	3	Molez	Barkedji	Senegal	2006	1994–1995	0.0874	2500	0	4	11	12	N	0.52
14	4,5	Mwangi	Ngerenya	Kenya	2003;2005	1999–2001	0.2231	10000	0	0.9	34	41	N	0.55
15	4,5	Mwangi	Chonyi	Kenya	2003;2005	1999–2001	0.3658	10000	0	0.9	50	76	N	0.60
16	4,5	Mwangi	Ngerenya	Kenya	2003;2005	1999–2001	0.2231	10000	1	5	188	546	N	0.74
17	4,5	Mwangi	Chonyi	Kenya	2003;2005	1999–2001	0.3658	10000	1	5	155	371	N	0.71
18	6	Owusu-Agyei	Kintampo	Ghana	2009	2004	0.6424	20000	0	0.9	100	45	N	0.31
19	6	Owusu-Agyei	Kintampo	Ghana	2009	2004	0.6424	20000	1	1.9	164	146	N	0.47
20	6	Owusu-Agyei	Kintampo	Ghana	2009	2004	0.6424	20000	2	2.9	138	108	N	0.44
21	6	Owusu-Agyei	Kintampo	Ghana	2009	2004	0.6424	20000	3	3.9	144	80	N	0.36
22	6	Owusu-Agyei	Kintampo	Ghana	2009	2004	0.6424	20000	4	4.9	87	46	N	0.35
23	7	Velema	Pahou	Benin	1991	1989	0.4551	20000	0	3	295	30	N	0.09
24	8	PRISM	Nagongera	Uganda	NA	2011	0.4740	5000	0	4	4	11	N	0.73
25	8	PRISM	Walukuba	Uganda	NA	2011	0.2488	5000	0	4	1	3	N	0.75

26	9	Afrane	Iguhu	Kenya	2014	2007-2009	0.0856	500	0	4	0.35	0.65	P	0.65

Additional Table 1 (Continued)

ID	Reference	Study	Site	Country	Publication Year	Year(s) studied	*Pf* PR_{0-5}	Parasite density threshold	AgeLow	AgeUp	*Pf* Pos under threshold	*Pf* Pos over threshold	Numeric or Proportion	Proportion over threshold
27	10	Cherif	Sapone	Burkina Faso	2017	2007	0.5566	5000	0.5	5	1301	1138	N	0.47
28	11	Mugyenyi	Ngerenya	Kenya	2017	2002	0.1220	2500	1	5	22	22	N	0.50
29	11	Mugyenyi	Ngerenya	Kenya	2017	2002-2003	0.1017	2500	1	5	34	99	N	0.74
30	11	Mugyenyi	Ngerenya	Kenya	2017	2003	0.1360	2500	1	5	36	61	N	0.63
31	12	Tiono	Banfora	Burkina Faso	2014	2009-2010	0.4776	5000	0	5	101	468	N	0.82

References:

1 Bloland PB, Boriga DA, Ruebush TK, *et al.* Longitudinal cohort study of the epidemiology of malaria infections in an area of intense malaria transmission II. Descriptive epidemiology of malaria infection and disease among children. *Am J Trop Med Hyg* 1999; 60: 641–8.

2 Lusingu JPA, Vestergaard LS, Mmbando BP, *et al.* Malaria morbidity and immunity among residents of villages with different *Plasmodium falciparum* transmission intensity in North-Eastern Tanzania. *Malar J* 2004; 3: 26.

3 Molez J, Diop A, Gaye O, Lemasson J, Fontenille D. Malaria morbidity in Barkedji, village of Ferlo, in Senegal Sahelian area. *Bull la Société Pathol Exot* 2006; 99: 187–90.

4 Mwangi TW, Ross A, Snow RW, Marsh K. Case Definitions of Clinical Malaria under Different Transmission Conditions in Kilifi District , Kenya. *J Infect Dis* 2005; 191: 1932–9.

5 Mwangi TW, Ross A, Marsh K, Snow RW. The effects of untreated bednets on malaria infection and morbidity on the Kenyan coast. *Trans R Soc Trop Med Hyg* 2003; 97: 369–72.

6 Owusu-agyei S, Asante KP, Adjuik M, *et al.* Epidemiology of malaria in the forest-savanna transitional zone of Ghana. *Malar J* 2009; 8: 220.

7 Velema JP, Alihonou EM, Chippaud J-P, Boxel Y van, Gbedj E, Adegbini R. Malaria morbidity and mortality in children under three years of age on the coast of Benin, West Africa. *Trans R Soc Trop Med Hyg* 1991; 85: 430–5.

8 PRISM dataset available at: https://clinepidb.org

9 Afrane YA, Zhou G, Githeko AK, Yan G. Clinical malaria case definition and malaria attributable fraction in the highlands of western Kenya. *Malar J* 2014; 13:405.

10 Cherif MK, Ouédraogo O, Sanou GS, *et al.* Antibody responses to *P . falciparum* blood stage antigens and incidence of clinical malaria in children living in endemic area in Burkina Faso. *BMC Res Notes* 2017; 10: 472.

11 Mugyenyi CK, Elliott SR, Yap XZ, *et al.* Declining Malaria Transmission Differentially Impacts the Maintenance of Humoral Immunity to *Plasmodium falciparum* in Children. *J Infect Dis* 2017; 216: 887–98.

12 Tiono AB, Kangoye DT, Rehman AM, *et al.* Malaria Incidence in Children in South-West Burkina Faso : Comparison of Active and Passive Case Detection Methods. *PLoS One* 2014; 9: e86936.
