# Supplementary material for: The contribution of non-malarial febrile illness co-infections to Plasmodium falciparum case counts in health facilities in sub-Saharan Africa
Source: Malar J. 2019 Jun 11;18:195. doi: 10.1186/s12936-019-2830-y (PMC6560910; doi:10.1186/s12936-019-2830-y)
Supplement: Supplementary file 2 — Additional file 2. Household survey data utilised for (i) modelling relationship between MAF and PfPR, and (ii) modelling proportion of MAF and NMFI cases seeking treatment in clinics. P+/− refers to the number of children P. falciparum positive/negative at time of interview. F+/− refers to the number of children for whom fever was/was not recalled in the 2 weeks preceding the survey. [file 12936_2019_2830_MOESM2_ESM.pdf]

Additional File 2. Household survey data utilised for *(i)* modelling relationship between MAF and *Pf* PR, and *(ii)* modelling proportion of MAF and NMFI cases seeking treatment in clinics. P+/- refers to the number of children *P. falciparum* positive/negative at time of interview. F+/- refers to the number of children for whom fever was/was not recalled in the two weeks preceding the survey.

Country	Survey start year	Survey start month	Survey end year	Survey end month	N	P+F+	P+F-	P-F+	P-F-	Sought treatment (public)	Sought treatment (any)
Angola	2006	12	2007	1	1156	73	210	170	703	93	119
Angola	2011	1	2011	6	2914	173	181	903	1657	554	610
Angola	2015	12	2016	1	3012	114	344	368	2186	146	258
Benin	2011	12	2012	1	3119	125	682	240	2072	99	158
Burkina Faso	2010	5	2010	12	5640	1020	3235	317	1068	699	744
Burkina Faso	2014	9	2014	12	5448	1768	1756	616	1308	1385	1468
Burundi	2012	12	2013	1	3465	473	226	1024	1742	730	870
Cameroon	2011	1	2011	8	4511	633	747	759	2372	310	777
Cote d'Ivoire	2011	12	2012	1	2638	345	828	359	1106	178	260
Democratic Republic of the Congo	2013	12	2014	1	6996	1029	1401	1288	3278	794	1194
Ghana	2014	9	2014	12	2341	210	734	178	1219	204	297
Ghana	2016	10	2016	11	1423	168	308	253	694	165	256
Guinea	2012	6	2012	10	2741	503	671	445	1122	267	383
Kenya	2015	7	2015	8	2845	153	85	982	1625	583	796
Liberia	2008	12	2009	1	3024	523	511	933	1057	548	1056
Liberia	2011	9	2011	12	2333	710	468	654	501	564	930
Liberia	2016	9	2016	11	2108	501	519	431	657	480	560
Madagascar	2011	3	2011	5	5212	113	210	748	4141	235	358
Madagascar	2013	4	2013	6	4476	79	199	537	3661	219	313
Madagascar	2016	4	2016	7	3139	34	71	459	2575	224	281
Malawi	2012	3	2012	4	1890	366	352	267	905	283	376
Malawi	2014	5	2014	6	1717	262	238	271	946	279	366
Mali	2012	12	2013	1	4108	231	1547	183	2147	111	168
Mali	2015	9	2015	11	6618	788	1132	1225	3473	688	928
Mozambique	2011	5	2011	12	4323	279	1188	330	2526	355	367
Mozambique	2015	6	2015	9	3709	493	715	580	1921	690	710
Nigeria	2010	10	2010	12	4302	905	1115	724	1558	447	1391

Additional File 3 (Continued)

Country	Survey start year	Survey start month	Survey end year	Survey end month	N	P+F+	P+F-	P-F+	P-F-	Sought treatment (public)	Sought treatment (any)
Nigeria	2015	10	2015	11	4920	1158	861	1048	1853	429	1427
Rwanda	2010	12	2011	1	3619	21	58	595	2945	302	338
Rwanda	2014	12	2015	1	3110	105	125	521	2359	338	386
Senegal	2008	12	2009	1	3519	176	248	1007	2088	408	518
Senegal	2010	12	2011	1	3278	27	81	695	2475	249	321
Senegal	2012	12	2013	1	5274	82	135	898	4159	391	489
Senegal	2015	2	2015	11	5441	21	37	928	4455	418	508
Tanzania	2007	12	2008	1	5576	240	456	792	4088	584	764
Tanzania	2011	12	2012	1	6379	249	331	1184	4615	685	1070
Tanzania	2015	12	2016	1	7366	291	564	1087	5424	209	1110
Togo	2013	12	2014	1	2844	309	731	367	1437	219	312
Uganda	2009	12	2010	2	3361	1037	710	570	1044	699	1319
Uganda	2014	12	2015	1	4022	642	655	682	2043	541	1037
Uganda	2016	6	2016	12	2753	583	291	532	1347	444	743

All surveys can be obtained with permission from the DHS Program: https://dhsprogram.com/
