# Supplementary material for: The contribution of non-malarial febrile illness co-infections to Plasmodium falciparum case counts in health facilities in sub-Saharan Africa
Source: Malar J. 2019 Jun 11;18:195. doi: 10.1186/s12936-019-2830-y (PMC6560910; doi:10.1186/s12936-019-2830-y)
Supplement: Supplementary file 3 — Additional file 3. Literature review and PRISM datasets utilised for modelling relationship between MAF and PfPR. For each study, the fever and parasite positivity status for each individual was extracted. Not all datapoints listed here were used in the final model; analysis was restricted to children under 5 years of age. *Search methodology for datapoint, as documented in “Methods”. **PRISM Data can be downloaded from the ClinEpiDb website: https://clinepidb.org/. [file 12936_2019_2830_MOESM3_ESM.pdf]

Additional File 3. Literature review and PRISM datasets utilised for modelling relationship between MAF and PfPR. For each study, the fever and parasite positivity status for each individual was extracted. Not all datapoints listed here were used in the final model; analysis was restricted to children under 5 years of age. *Search methodology for datapoint, as documented in Methods. ** PRISM Data can be downloaded from the ClinEpiDb website: https://clinepidb.org/

ID	Reference	Point used in model?	Source *	Author	Publication Year	Study Year	AgeLow	AgeUp	Method for P+	Fever recall or measured?	Fever period
1	1	No	MAP PfPR database	Erhardt	2006	2002	0	4	PCR	Measured	-
2	1	No	MAP PfPR database	Erhardt	2006	2002	5	9	PCR	Measured	-
3	1	No	MAP PfPR database	Erhardt	2006	2002	0	4	PCR	Measured	-
4	1	No	MAP PfPR database	Erhardt	2006	2002	5	9	PCR	Measured	-
5	2	No	MAP PfPR database	Schellenberg	1994	1989	1	5	Microscopy	Measured	-
6	3	No	MAP PfPR database	Bisoffi	2010	2006	0	99	Microscopy	Measured	-
7	3	Yes	MAP PfPR database	Bisoffi	2010	2006	0	1.00	Microscopy	Measured	-
8	3	Yes	MAP PfPR database	Bisoffi	2010	2006	1	4	Microscopy	Measured	-
9	3	No	MAP PfPR database	Bisoffi	2010	2006	5	14	Microscopy	Measured	-
10	3	No	MAP PfPR database	Bisoffi	2010	2006	15	99	Microscopy	Measured	-
11	4	Yes	MAP PfPR database	Mabunda	2009	2002-2003	0	1.00	Microscopy	Measured	-
12	4	Yes	MAP PfPR database	Mabunda	2009	2002-2003	1	1	Microscopy	Measured	-
13	4	Yes	MAP PfPR database	Mabunda	2009	2002-2003	2	4	Microscopy	Measured	-
14	4	No	MAP PfPR database	Mabunda	2009	2002-2003	5	6	Microscopy	Measured	-
15	4	No	MAP PfPR database	Mabunda	2009	2002-2003	7	9	Microscopy	Measured	-
16	5	No	MAP PfPR database	Mayor	2007	1999	15	83	Microscopy	Recall	24 hours
17	6	No	MAP PfPR database	Moukandja	2016	2013 (April)	3	85	Microscopy	Measured	-
18	6	No	MAP PfPR database	Moukandja	2016	2013 (June)	3	85	Microscopy	Measured	-
19	6	No	MAP PfPR database	Moukandja	2016	2013 (July)	3	85	Microscopy	Measured	-
20	6	No	MAP PfPR database	Moukandja	2016	2013 (October)	3	85	Microscopy	Measured	-
21	6	No	MAP PfPR database	Moukandja	2016	2014 (March)	3	85	Microscopy	Measured	-
22	6	No	MAP PfPR database	Moukandja	2016	2014 (June)	3	85	Microscopy	Measured	-
23	7	No	MAP PfPR database	Barbosa	2014	2010 (March - May)	0.25	99	Microscopy	Recall	7 days
24	7	No	MAP PfPR database	Barbosa	2014	2010 (May - July)	0.25	99	Microscopy	Recall	7 days
25	7	No	MAP PfPR database	Barbosa	2014	2010 (October - Nov)	0.25	99	Microscopy	Recall	7 days
26	7	No	MAP PfPR database	Barbosa	2014	2011 (March - April)	0.25	99	Microscopy	Recall	7 days
27	7	No	MAP PfPR database	Barbosa	2014	2011 (October - Nov)	0.25	99	Microscopy	Recall	7 days
28	7	No	MAP PfPR database	Barbosa	2014	2012 (April - May)	0.25	99	Microscopy	Recall	7 days
29	7	No	MAP PfPR database	Barbosa	2014	2012 (October Nov)	0.25	99	Microscopy	Recall	7 days

Additional File 4 (Continued)

ID	Reference	Point used in model?	Source *	Author	Publication Year	Study Year	AgeLow	AgeUp	Method for P+	Fever recall or measured?	Fever period
30	7	No	MAP PfPR database	Barbosa	2014	2013 (April - May)	0.25	99	Microscopy	Recall	7 days
31	7	No	MAP PfPR database	Barbosa	2014	2010 (March - May)	0.25	99	PCR	Recall	7 days
32	7	No	MAP PfPR database	Barbosa	2014	2010 (May - July)	0.25	99	PCR	Recall	7 days
33	7	No	MAP PfPR database	Barbosa	2014	2010 (October - Nov)	0.25	99	PCR	Recall	7 days
34	7	No	MAP PfPR database	Barbosa	2014	2011 (March - April)	0.25	99	PCR	Recall	7 days
35	7	No	MAP PfPR database	Barbosa	2014	2011 (October - Nov)	0.25	99	PCR	Recall	7 days
36	7	No	MAP PfPR database	Barbosa	2014	2012 (April - May)	0.25	99	PCR	Recall	7 days
37	7	No	MAP PfPR database	Barbosa	2014	2012 (October-Nov)	0.25	99	PCR	Recall	7 days
38	7	No	MAP PfPR database	Barbosa	2014	2013 (April - May)	0.25	99	PCR	Recall	7 days
39	8	Yes	MAP PfPR database	Burdam	2016	2013	1	4	Microscopy	Recall	24 hours
40	9	No	MAP PfPR database	Imwong	2015	2013	0	99	PCR	Recall	Unknown
41	10	No	MAP PfPR database	Phommasone	2016	2015	15	80	PCR	Recall	2 days
42	11	No	MAP PfPR database	Geiger	2013	2009-2012	0.5	90	Microscopy	Measured	-
43	12	No	MAP PfPR database	Mboera	2015	2012	4	16	Microscopy	Measured	-
44	13	No	MAP PfPR database	Mwesigwa	2015	2012	0	99	PCR	Recall	24 hours
45	13	No	MAP PfPR database	Mwesigwa	2015	2012	0	99	PCR	Measured	-
46	14	No	MAP PfPR database	Ouattara	2014	2010-2012	0	15	RDT	Measured	-
47	14	No	MAP PfPR database	Ouattara	2014	2010-2012	0	15	Microscopy	Measured	-
48	15	No	MAP PfPR database	Charlwood	2015	2007	0	75	Microscopy	Measured	-
49	15	No	MAP PfPR database	Charlwood	2015	2009	0	75	Microscopy	Measured	-
50	15	No	MAP PfPR database	Charlwood	2015	2010	0	75	Microscopy	Measured	-
51	15	No	MAP PfPR database	Charlwood	2015	2011	0	75	Microscopy	Measured	-
52	16	No	MAP PfPR database	Kimbi	2013	2011	4	15	Microscopy	Measured	-
53	17	No	MAP PfPR database	Mathanga	2015	2011	5	21	Microscopy	Recall	14 days
54	17	No	MAP PfPR database	Mathanga	2015	2011	5	21	Microscopy	Measured	-
55	18	No	MAP PfPR database	Gneme	2013	2007-2010	3	15	Microscopy	Measured	-
56	19	No	MAP PfPR database	Proietti	2011	2009	0	9	Microscopy	Measured	-
57	20	No	MAP PfPR database	Sutcliffe	2011	2007-2009	0	99	RDT	Measured	-
58	20	No	MAP PfPR database	Sutcliffe	2011	2007-2009	0	99	RDT	Recall	2 days
59	21	No	MAP PfPR database	Gitonga	2010	2008-2010	5	20	RDT	Measured	-
60	22	No	MAP PfPR database	Damien	2010	2007-2008	0	5	Microscopy	Measured	-

Additional File 4 (Continued)

ID	Reference	Point used in model?	Source *	Author	Publication Year	Study Year	AgeLow	AgeUp	Method for P+	Fever recall or measured?	Fever period
61	23	No	MAP PfPR database	Eisele	2007	2006	0	92	PCR	Measured	-
62	24	No	MAP PfPR database	Mustafa	2009	2005-2006	1	70	Microscopy	Measured	-
63	24	No	MAP PfPR database	Mustafa	2009	2005-2006	1	73	Microscopy	Measured	-
64	24	No	MAP PfPR database	Mustafa	2009	2005-2006	1	99	Microscopy	Measured	-
65	24	No	MAP PfPR database	Mustafa	2009	2005-2006	1	90	Microscopy	Measured	-
66	25	Yes	MAP PfPR database	Ouédraogo	2008	2006	0.5	1	Microscopy	Measured	-
67	26	No	MAP PfPR database	Pluess	2009	2006	0	99	Microscopy	Measured	-
68	26	No	MAP PfPR database	Pluess	2009	2006	0	99	Microscopy	Recall	14 days
69	27	No	PubMed search	Tchuinkam	2015	Unknown	0	99	Microscopy	Measured	-
70	27	No	PubMed search	Tchuinkam	2015	Unknown	0	99	Microscopy	Measured	-
71	28	No	PubMed search	Incardona	2007	2001	0	99	Microscopy	Measured	-
72	28	No	PubMed search	Incardona	2007	2002	0	99	Microscopy	Measured	-
73	28	No	PubMed search	Incardona	2007	2003	0	99	Microscopy	Measured	-
74	29	Yes	PubMed search	Dicko	2005	1993	0.5	0.99	Microscopy	Measured	-
75	29	Yes	PubMed search	Dicko	2005	1993	1	1	Microscopy	Measured	-
76	29	Yes	PubMed search	Dicko	2005	1993	2	4	Microscopy	Measured	-
77	29	No	PubMed search	Dicko	2005	1993	5	9	Microscopy	Measured	-
78	29	Yes	PubMed search	Dicko	2005	1993	0.5	0.99	Microscopy	Measured	-
79	29	Yes	PubMed search	Dicko	2005	1993	1	1	Microscopy	Measured	-
80	29	Yes	PubMed search	Dicko	2005	1993	2	4	Microscopy	Measured	-
81	29	No	PubMed search	Dicko	2005	1993	5	9	Microscopy	Measured	-
82	30	Yes	PubMed search	Vounatsou	1999	1992-1993	0	0.17	Microscopy	Measured OR recall	Measured/24h recall
83	30	Yes	PubMed search	Vounatsou	1999	1992-1993	0.25	0.42	Microscopy	Measured OR recall	Measured/24h recall
84	30	Yes	PubMed search	Vounatsou	1999	1992-1993	0.5	0.67	Microscopy	Measured OR recall	Measured/24h recall
85	30	Yes	PubMed search	Vounatsou	1999	1992-1993	0.75	0.92	Microscopy	Measured OR recall	Measured/24h recall
86	30	Yes	PubMed search	Vounatsou	1999	1992-1993	0	0.17	Microscopy	Measured OR recall	Measured/24h recall
87	30	Yes	PubMed search	Vounatsou	1999	1992-1993	0.25	0.42	Microscopy	Measured OR recall	Measured/24h recall
88	30	Yes	PubMed search	Vounatsou	1999	1992-1993	0.5	0.67	Microscopy	Measured OR recall	Measured/24h recall
89	30	Yes	PubMed search	Vounatsou	1999	1992-1993	0.75	0.92	Microscopy	Measured OR recall	Measured/24h recall
90	31	No	PubMed search	Charchuk	2016	2013	0	2	RDT	Recall	2 weeks
91	31	No	PubMed search	Charchuk	2016	2013	3	5	RDT	Recall	2 weeks

Additional File 4 (Continued)

ID	Reference	Point used in model?	Source *	Author	Publication Year	Study Year	AgeLow	AgeUp	Method for P+	Fever recall or measured?	Fever period
92	31	No	MAP PfPR database	Charchuk	2016	2012	5	19	RDT	Measured	NA
93	32	No	MAP PfPR database	Parr	2016	2011-2013	0	99	Microscopy and RDT	Recall	24h
94	33	No	MAP PfPR database	Pava	2016	2013	0	99	Microscopy	Recall	30 days
95	34	Yes	PRISM Data	-	-	2011	0	4	Microscopy	Measured	NA
96	34	Yes	PRISM Data	-	-	2011	0	4	Microscopy	Measured	NA
97	34	Yes	PRISM Data	-	-	2011	0	4	Microscopy	Measured	NA
98	34	Yes	PRISM Data	-	-	2011	0	4	Microscopy	Recall	24h
99	34	Yes	PRISM Data	-	-	2011	0	4	Microscopy	Recall	24h
100	34	Yes	PRISM Data	-	-	2011	0	4	Microscopy	Recall	24h
101	34	No	PRISM Data	-	-	2011	5	14	Microscopy	Measured	NA
102	34	No	PRISM Data	-	-	2011	5	14	Microscopy	Measured	NA
103	34	No	PRISM Data	-	-	2011	5	14	Microscopy	Measured	NA
104	34	No	PRISM Data	-	-	2011	5	14	Microscopy	Recall	24h
105	34	No	PRISM Data	-	-	2011	5	14	Microscopy	Recall	24h
106	34	No	PRISM Data	-	-	2011	5	14	Microscopy	Recall	24h
107	34	No	PRISM Data	-	-	2011	15	71	Microscopy	Measured	NA
108	34	No	PRISM Data	-	-	2011	15	71	Microscopy	Measured	NA
109	34	No	PRISM Data	-	-	2011	15	71	Microscopy	Measured	NA
110	34	No	PRISM Data	-	-	2011	15	71	Microscopy	Recall	24h
111	34	No	PRISM Data	-	-	2011	15	71	Microscopy	Recall	24h
112	34	No	PRISM Data	-	-	2011	15	71	Microscopy	Recall	24h

Additional File 4 (Continued)

ID	Reference	N	P+F+	P+F-	P-F+	P-F-	P+	F+	Estimated MAF cases	Estimated MAF cases as a proportion of P+F+
1	1	1267	34	18	54	1161	52	88	31.69	0.93
2	1	842	24	6	10	802	30	34	23.63	0.98
3	1	1104	72	10	23	999	82	95	70.15	0.97
4	1	1015	32	5	8	970	37	40	31.70	0.99
5	2	407	41	86	33	247	127	74	26.03	0.63
6	3	1426	147	121	519	639	268	666	26.89	0.18
7	3	209	132	18	27	32	150	159	63.36	0.48
8	3	665	394	72	110	89	466	504	136.41	0.35
9	3	412	198	72	77	65	270	275	51.59	0.26
10	3	969	117	153	262	437	270	379	15.80	0.14
11	4	1517	161	479	68	809	640	229	111.38	0.69
12	4	1609	166	725	46	672	891	212	108.92	0.66
13	4	3515	162	1641	88	1624	1803	250	69.32	0.43
14	4	1244	40	558	33	613	598	73	9.45	0.24
15	4	951	31	343	19	558	374	50	18.68	0.60
16	5	249	6	30	33	180	36	39	0.42	0.07
17	6	370	8	153	1	208	161	9	7.23	0.90
18	6	267	1	108	0	158	109	1	1.00	1.00
19	6	218	2	113	2	101	115	4	0.00	0.00
20	6	255	1	86	0	168	87	1	1.00	1.00
21	6	232	9	125	5	93	134	14	2.16	0.24
22	6	145	1	66	0	78	67	1	1.00	1.00
23	7	159	0	1	62	96	1	63	0.00	0.00
24	7	199	0	1	53	145	1	54	0.00	0.00
25	7	211	0	0	45	166	0	45	0.00	0.00
26	7	216	0	0	34	182	0	34	0.00	0.00
27	7	204	0	0	40	164	0	40	0.00	0.00
28	7	178	0	0	22	156	0	22	0.00	0.00
29	7	199	0	0	65	134	0	65	0.00	0.00

Additional File 4 (Continued)

ID	Reference	N	P+F+	P+F-	P-F+	P-F-	P+	F+	Estimated MAF cases	Estimated MAF cases as a proportion of P+F+
30	7	175	0	0	46	129	0	46	0.00	0.00
31	7	150	3	7	59	81	10	62	0.00	0.00
32	7	187	1	11	51	124	12	52	0.00	0.00
33	7	198	4	20	38	136	24	42	0.00	0.00
34	7	199	1	1	30	167	2	31	0.70	0.70
35	7	197	0	0	38	159	0	38	0.00	0.00
36	7	177	0	0	21	156	0	21	0.00	0.00
37	7	199	0	0	65	134	0	65	0.00	0.00
38	7	169	0	0	43	126	0	43	0.00	0.00
39	8	533	3	19	3	508	22	6	2.87	0.96
40	9	1465	43	185	139	1098	228	182	17.38	0.40
41	10	858	21	25	249	563	46	270	6.89	0.33
42	11	1764	199	552	164	849	751	363	77.42	0.39
43	12	1019	2	44	12	961	46	14	1.43	0.72
44	13	9094	711	745	3484	4154	1456	4195	46.86	0.07
45	13	9094	209	1247	289	7349	1456	498	153.91	0.74
46	14	1482	249	698	147	388	947	396	0.00	0.00
47	14	1482	236	718	160	368	954	396	0.00	0.00
48	15	32	7	0	3	22	7	3	6.16	0.88
49	15	431	7	153	7	264	160	14	2.87	0.41
50	15	369	19	82	25	243	101	44	9.58	0.50
51	15	258	3	119	2	134	122	5	1.21	0.40
52	16	728	88	158	130	352	246	218	21.65	0.25
53	17	2645	541	1046	426	632	1587	967	0.00	0.00
54	17	2641	449	1134	287	771	1583	736	19.59	0.04
55	18	830	111	413	45	261	524	156	33.94	0.31
56	19	425	85	168	42	130	253	127	23.22	0.27
57	20	330	8	71	6	245	79	14	6.11	0.76
58	20	330	41	38	82	169	79	123	15.19	0.37
59	21	30542	55	1258	678	28551	1313	733	24.54	0.45
60	22	3074	105	618	131	2220	723	236	64.71	0.62

Additional File 4 (Continued)

ID	Reference	N	P+F+	P+F-	P-F+	P-F-	P+	F+	Estimated MAF cases	Estimated MAF cases as a proportion of P+F+
61	23	708	9	14	52	633	23	61	7.25	0.81
62	24	421	21	4	47	349	25	68	18.03	0.86
63	24	522	68	17	45	392	85	113	59.25	0.87
64	24	1049	40	20	99	890	60	139	33.99	0.85
65	24	1636	45	2	53	1536	47	98	43.43	0.97
66	25	456	123	117	79	137	240	202	35.22	0.29
67	26	723	8	234	12	469	242	20	1.96	0.25
68	26	723	70	172	116	365	242	186	11.64	0.17
69	27	675	243	98	166	168	341	409	73.52	0.30
70	27	736	126	43	171	396	169	297	75.03	0.60
71	28	4074	35	88	166	3785	123	201	29.83	0.85
72	28	2624	42	142	373	2067	184	415	13.87	0.33
73	28	4954	199	411	404	3940	610	603	142.27	0.71
74	29	162	29	54	7	72	83	36	21.65	0.75
75	29	231	38	110	6	77	148	44	27.30	0.72
76	29	811	93	462	26	230	555	119	36.63	0.39
77	29	1230	105	692	49	384	797	154	14.81	0.14
78	29	77	3	15	4	55	18	7	1.78	0.59
79	29	313	13	93	11	196	106	24	7.37	0.57
80	29	772	35	342	25	370	377	60	11.14	0.32
81	29	1191	60	482	39	610	542	99	27.43	0.46
82	30	203	15	48	16	124	63	31	7.80	0.52
83	30	202	40	81	18	63	121	58	13.11	0.33
84	30	140	35	56	13	36	91	48	10.86	0.31
85	30	101	30	41	3	27	71	33	22.90	0.76
86	30	189	11	25	20	133	36	31	6.29	0.57
87	30	209	33	75	17	84	108	50	14.82	0.45
88	30	177	44	74	9	50	118	53	26.00	0.59
89	30	135	31	68	9	27	99	40	6.25	0.20
90	31	233	5	20	57	151	25	62	0.00	0.00
91	31	167	4	24	34	105	28	38	0.00	0.00

Additional File 4 (Continued)

ID	Reference	N	P+F+	P+F-	P-F+	P-F-	P+	F+	Estimated MAF cases	Estimated MAF cases as a proportion of P+F+
92	31	109	5	39	7	58	44	12	0.26	0.05
93	32	800	17	63	91	629	80	108	6.89	0.41
94	33	2761	8	137	102	2514	145	2616	2.35	0.29
95	34	211	1	14	13	183	15	14	0.01	0.01
96	34	215	15	43	16	141	58	31	9.09	0.61
97	34	222	4	12	15	191	16	19	2.83	0.71
98	34	211	4	11	13	183	15	17	3.01	0.75
99	34	215	19	39	26	131	58	45	9.39	0.49
100	34	222	3	13	16	190	16	19	1.76	0.59
101	34	169	3	10	6	150	13	9	2.50	0.83
102	34	149	4	56	4	85	60	8	1.30	0.33
103	34	118	0	5	7	106	5	7	-0.31	0.00
104	34	169	5	8	5	151	13	10	4.58	0.92
105	34	149	5	55	7	82	60	12	0.28	0.06
106	34	118	0	5	6	107	5	6	-0.27	0.00
107	34	98	0	0	5	93	0	5	0.00	0.00
108	34	106	1	4	4	97	5	5	0.80	0.80
109	34	114	1	4	6	103	5	7	0.72	0.72
110	34	98	0	0	2	96	0	2	0.00	0.00
111	34	106	1	4	7	94	5	8	0.65	0.65
112	34	114	1	4	3	106	5	4	0.86	0.86

Additional File 4 (Continued)

Reference

1 Ehrhardt S, Burchard GD, Mantel C, et al. Malaria, anemia, and malnutrition in African children--defining intervention priorities. J Infect Dis 2006; 194: 108–14.

2 Schellenberg J, Smith T, Alonso P, et al. What is Clinical Malaria? Finding Case Definitions for Field Research in Highly Endemic Areas. Parasitol Today 1994; 10: 439–42.

3 Bisoffi Z, Sirima SB, Menten J, et al. Accuracy of a rapid diagnostic test on the diagnosis of malaria infection and of malaria-attributable fever during low and high transmission season in Burkina Faso. Malar J 2010; 9: 192.

4 Mabunda S, Aponte JJ, Tiago A, Alonso P. A country-wide malaria survey in Mozambique. II. Malaria attributable proportion of fever and establishment of malaria case definition in children across different epidemiological settings. Malar J 2009; 8: 74.

5 Mayor A, Aponte JJ, Fogg C, et al. The epidemiology of malaria in adults in a rural area of southern Mozambique. Malar J 2007; 6: 3.

6 Moukandja IP, Essone JCBB, Sagara I, et al. Marked Rise in the Prevalence of Asymptomatic Plasmodium falciparum Infection in Rural Gabon. PLoS One 2016; 11: e0153899.

7 Barbosa S, Gozze A, Lima N, et al. Epidemiology of Disappearing *Plasmodium vivax* Malaria: A Case Study in Rural Amazonia. PLoS Negl Trop Dis 2014; 8.

8 Burdam FH, Hakimi M, Thio F, et al. Asymptomatic Vivax and Falciparum Parasitaemia with Helminth Co-Infection: Major Risk Factors for Anaemia in Early Life. PLoS One 2016; 11: e0160917.

9 Imwong M, Nguyen TN, Tripura R, et al. The epidemiology of subclinical malaria infections in South-East Asia: findings from cross-sectional surveys in Thailand – Myanmar border areas, Cambodia, and Vietnam. Malar J 2015; 14:381.

10 Phommasone K, Adhikari B, Henriques G, et al. Asymptomatic *Plasmodium* infections in 18 villages of southern Savannakhet Province, Lao PDR (Laos). Malar J 2016; 15:296.

11 Geiger C, Agustar HK, Compaoré G, et al. Declining malaria parasite prevalence and trends of asymptomatic parasitaemia in a seasonal transmission setting in north-western Burkina Faso between 2000 and 2009 – 2012. Malar J 2013; 12:27

12 Mboera LEG, Bwana VM, Rumisha SF, et al. Malaria, anaemia and nutritional status among schoolchildren in relation to ecosystems, livelihoods and health systems in Kilosa District in central Tanzania. BMC Public Health 2015; 15:553.

13 Mwesigwa J, Okebe J, Affara M, et al. On-going malaria transmission in The Gambia despite high coverage of control interventions: a nationwide cross-sectional survey. Malar J 2015; 14:314.

14 Ouattara AF, Dagnogo M, Olliaro PL, et al. *Plasmodium falciparum* infection and clinical indicators in relation to net coverage in central Côte d'Ivoire. Parasit Vectors 2014; 7:306.

15 Charlwood JD, Tom EVE, Cuamba N, Alifrangis M, Stanton M. Malaria prevalence and incidence in an isolated , meso-endemic area of Mozambique. PeerJ 2015; 3: e1370.

16 Kimbi HK, Sumbele IUN, Nweboh M, et al. Malaria and haematologic parameters of pupils at different altitudes along the slope of Mount Cameroon: a cross-sectional study. Malar J 2013; 12: 193.

17 Mathanga DP, Halliday KE, Jawati M, et al. The High Burden of Malaria in Primary School Children in Southern Malawi. Am J Trop Med Hyg 2015; 93: 779–89.

18 Gnémé A, Guelbéogo WM, Riehle MM, et al. *Plasmodium* species occurrence , temporal distribution and interaction in a child-aged population in rural Burkina Faso. Malar J 2013; 12: 67.

Additional File 4 (Continued)

Reference

19 Proietti C, Pettinato DD, Kanoi BN, et al. Continuing Intense Malaria Transmission in Northern Uganda. Am J Trop Med Hyg 2011; 84: 830–7.

20 Sutcliffe CG, Kobayashi T, Hamapumbu H, et al. Changing individual-level risk factors for malaria with declining transmission in southern Zambia : a cross-sectional study. Malar J 2011; 10: 324.

21 Gitonga CW, Karanja PN, Kihara J, et al. Implementing school malaria surveys in Kenya: towards a national surveillance system. Malar J 2010; 9: 306.

22 Damien GB, Djènontin A, Rogier C, et al. Malaria infection and disease in an area with pyrethroid-resistant vectors in southern Benin. Malar J 2010; 9: 380.

23 Eisele TP, Keating J, Bennett A, et al. Prevalence of *Plasmodium falciparum* Infection in Rainy Season, Artibonite Valley, Haiti. Emerg Infect Dis 2007; 13: 1494–6.

24 Mustafa HS, Malik EM, Tuok HT, Mohamed AA, Julla AI, Bassili A. Malaria preventive measures , health care seeking behaviour and malaria burden in different epidemiological settings in Sudan. Trop Med Int Heal 2009; 14: 1488–95.

25 Ouedraogo H, Zeba A, Dramaix-Wilmet M, Donnen P. Moderate-to-Severe Anaemia due to Afebrile *Plasmodium falciparum* Infection in Children aged 6 – 23 Months from the Rural District of Kongoussi , Burkina Faso. J Trop Pediatr 2008; 54: 395–400.

26 Pluess B, Mueller I, Levi D, King G, Smith TA, Lengeler C. Malaria – a major health problem within an oil palm plantation around Popondetta, Papua New Guinea. Malar J 2009; 8: 56.

27 Tchuinkam T, Nyih-kong B, Fopa F, et al. Distribution of *Plasmodium falciparum* gametocytes and malaria-attributable fraction of fever episodes along an altitudinal transect in Western Cameroon. Malar J 2015; 14:96.

28 Incardona S, Vong S, Chiv L, et al. Large-scale malaria survey in Cambodia: novel insights on species distribution and risk factors. Malar J 2007; 6: 37.

29 Dicko A, Mantel C, Kouriba B, Sagara I, Thera MA, Doumbia S. Season, fever prevalence and pyrogenic threshold for malaria disease definition in an endemic area of Mali. Trop Med Int Heal 2005; 10: 550–6.

30 Vounatsou P, Smith T, Kitua AY, Alonso PL, Tanner M. Apparent tolerance of *Plasmodium falciparum* in infants in a highly endemic area. Parasitology 2000; 120: 1–9.

31 Charchuk R, Katsuva M, Paul J, Claude KM, Houston S, Hawkes MT. Burden of malaria is higher among children in an internal displacement camp compared to a neighbouring village in the Democratic Republic of the Congo. Malar J 2016; 15. DOI:10.1186/s12936-016-1479-z

32 Parr, JB, Benson, C, Patel, JC, Hoffman, IF , Kamthunzi, P, Martinson, F and Tegha, G. (2016). Estimation of *Plasmodium falciparum* Transmission Intensity in Lilongwe, Malawi, by Microscopy, Rapid Diagnostic Testing, and Nucleic Acid Detection. American Journal of Tropical Medicine and Hygiene, 95(2):373-377

33 Pava, Z., Burdam, F.H., Handayuni, I., Trianty, L., Utami, R.A.S., Tirta, Y.K., Kenangalem, E., Lampah, D., Kusuma, A., Wirjanata, G., Kho, S., Simpson, J.A., Auburn, S., Douglas, N.M., Noviyanti, R., Anstey, N.M., Poespoprodjo, J.R., Marfurt, J. and Price, R.N. (2016). Submicroscopic and Asymptomatic *Plasmodium* Parasitaemia Associated with Significant Risk of Anaemia in Papua, Indonesia. PloS One, 11(10):e0165340

34 PRISM **
