# Supplementary material for: The contribution of non-malarial febrile illness co-infections to Plasmodium falciparum case counts in health facilities in sub-Saharan Africa
Source: Malar J. 2019 Jun 11;18:195. doi: 10.1186/s12936-019-2830-y (PMC6560910; doi:10.1186/s12936-019-2830-y)
Supplement: Supplementary file 4 — Additional file 4. Datasets and modelling approach for estimation of the relationship between malaria-attributable fever and Plasmodium falciparum prevalence in children under 5 years of age. [file 12936_2019_2830_MOESM4_ESM.pdf]

Additional File 4. Datasets and modelling approach for estimation of the relationship between malaria-attributable fever and *Plasmodium falciparum* prevalence in children under five years of age

1. Datasets

In order to estimate the relationship between malaria-attributable fever (MAF) and *Plasmodium falciparum* prevalence in children under five years of age (*Pf*PR$_{0\text{-}5}$), four primary datasets were assembled. These datasets were chosen to encompass the variety of fever definitions and methods for defining malaria-attributable fever. The datasets fell broadly into two categories: defining MAF using a pyrogenic parasite density threshold for malaria-infected individuals, above which malaria-positive fevers are defined as MAF (one dataset); and datasets which record paired observations of malaria positivity and fever (either measured fever at the point of diagnosis, or recalled recent fever history) allowing for empirical estimation of MAF (three datasets).

Dataset 1: estimating MAF using a parasite-density threshold for malaria-infected individuals

Estimates of the proportion of malaria infections above and below a pyrogenic parasite density threshold were derived from active case detection studies. Analysis was restricted only to active case detection studies as these fully enumerate all febrile illnesses within the study site. The parasite density threshold dataset comprised a subset of the exhaustive dataset of *P. falciparum* active case detection studies compiled by Battle *et al,*[1] updated to include all studies published until the end of 2017. The restricted set of studies included active case detection studies, where blood parasite densities of infected individuals were collated, and matched, site-specific, age-appropriate *Pf*PR was recorded over the longitudinal study. Full inclusion criteria for this analysis were as follows: *i)* parasite densities of symptomatic individuals were given (either as raw values or above/below a case definition parasite density threshold; for studies that presented multiple case definition thresholds the threshold closest to 5,000 parasites/µl was used), *ii)* the study reported only clinical incidence amongst children between 0 and 5 years of age. For each study, the total number of microscopically-diagnosed *P. falciparum* positive febrile individuals above and below the malaria case definition threshold of 5,000 parasites/µl was collated, for consistency with previous models.[2] A total of 11 studies met the inclusion criteria, resulting in 31 active case detection records. Full details of the studies utilised are given in Additional File 2.

Datasets 2-4: Empirical estimation of MAF

Datasets 2-4 consisted of paired observations of malaria and fever positivity, whereby the proportion of malaria-attributable fevers within malaria-positive fevers was derived empirically. Under this framework, the number of malaria-attributable fevers within a study site, N_{MAF}, is given by

$$N_{MAF} = N_{P^+F^+} - (N_{P^+} \times \frac{N_{P^-F^+}}{N_{P^-}})$$

Where $N_{P^+F^+}$ is the number of individuals who are both *P. falciparum*-positive and fever-positive, N_{P^+} is the total number of *P. falciparum*-positive individuals, $N_{P^-F^+}$ is the total number of individuals who are both *P. falciparum*-negative and fever-positive, and N_{P^-} is the total number of *P. falciparum*-negative individuals. This framework can be applied to any study site where paired observations of fever and malaria are available. In this analysis, three datasets were compiled and used for empirical estimation of MAF. These datasets are labelled "datasets 2-4" and are described in turn below.

Dataset 2: Household survey data

This dataset was used for empirical estimation of MAF and was composed of 41 cross-sectional, nationally-representative georeferenced surveys of malaria prevalence in children less than five years of age across 21 countries conducted between 2006-2016 in sub-Saharan Africa, obtained from the Demographic and Health (DHS) Program.[3] Full details of the surveys used can be found in Additional File 3. In each of these surveys,

interviewers visited houses (selected as a geographically and demographically representative sample of the population) and a finger- or heel-prick blood sample was taken from any children under five years of age present and tested for malaria with an RDT. For each child receiving a malaria diagnosis, their caregiver was asked whether, in the past two weeks, the child had a fever. Diagnostic and fever history outcomes were collated from each child, with children aggregated at geo-located cluster-level to represent community-level groups, resulting in a total of 156,670 paired observations of malaria positivity and fever history in the same individuals. A limitation of the household survey data is that an individual who was malaria-positive at a previous point during the two weeks preceding the interview may have sought and received antimalarial treatment, and their blood antigen concentration may have reduced sufficiently to appear RDT-negative by the time of interview (referred to as the "treatment effect" in the main manuscript). The treatment effect is accounted for in both models described in subsequent sections.

Dataset 3: Literature review

This dataset was used for empirical estimation of MAF and was composed of paired observations of malaria positivity and fever collated from published articles. Two search strategies were utilised to identify these studies. First, every article published between 2006 and 2017 cited within the Malaria Atlas Project database (the largest repository of global malaria prevalence data, updated annually via exhaustive reviews of published literature),[4] was systematically searched for paired observations of fever and malaria positivity. Second, a systematic literature review of the PubMed database using the MeSh terms "malaria", "fever" and "diagnosis" was conducted. For both data sources, studies assessing a population in a malaria-endemic community were collated, using the following inclusion criteria: *i)* malaria diagnosis was conducted (using any diagnostic), and *ii)* diagnostic outcomes were paired with individual fever status, whether measured at the time of diagnosis or individually recalled recent fever history. For each study meeting the inclusion criteria, the location of study was recorded and the number of individuals in each community falling in to each of the following four categories was documented: *i) P. falciparum* positive and fever positive, *ii), P. falciparum* positive and fever negative, *iii) P. falciparum* negative and fever positive, and *iv) P. falciparum* negative and fever negative. The review of the MAP *Pf*PR database and the PubMed review yielded 71 and 23 observations, respectively, of community-level paired malaria and fever measurements, comprising a total of 123,649 individuals.

Dataset 4: *Program for Resistance, Immunology, Surveillance and Modeling of Malaria in Uganda* (PRISM) study

The final database utilised for empirical estimates of MAF was sourced from the *Program for Resistance, Immunology, Surveillance and Modeling of Malaria in Uganda* (PRISM) study.[5] The PRISM study collected data over five years (2011-2016) on malaria-related metrics across three sites of differing malaria transmission intensity in Uganda, with the aim of improving understanding of the complex relationships between malaria parasite, host, and vector. In each of the three Ugandan sites, approximately 100 households were enrolled between 2011 and 2013 and followed up longitudinally for malaria infection. The malaria diagnostic outcome of all individuals at enrolment was collated, and paired with measured fever (axillary temperature > 38°C) at the time of diagnosis.

2. Modelling approach for estimating relationship between MAF and *Pf*PR$_{0\text{-}5}$

For this analysis, a model was applied where the relationship between MAF in children under 5 years of age and *Pf*PR$_{0\text{-}5}$ was learned. The DHS Program data (described above as Dataset 2) was used to inform the shape of the relationship, and the remaining three datasets described above (Datasets 1, 3 and 4) were subsequently used to learn scaling factors, as these datasets are informative for higher values of *Pf*PR$_{0\text{-}5}$ but lack MAF observations where *Pf*PR$_{0\text{-}5}$ <0.1. Additionally these datasets were unbiased by the "treatment effect" of DHS Program data, described in the previous sub-section.

We assume this relationship takes the following form, the motivation for which was informed by localised regression fits to the response data detailed above:

$$p_{MAF} = \beta\left(e^{-\lambda \times p_{pos}} + \alpha\right)$$

Where

$$p_{MAF} = \frac{N_{MAF}}{N_{MF}}$$

Where N_{MAF} is the number of individuals in a given population with a malaria-attributable fever, N_{MF} is the number of individuals in a given population with a malaria-positive fever (regardless of fever causality) and p_{MAF} is the proportion of malaria-positive fevers that are causally due to malaria. It was assumed that this proportion is negatively exponential and is shaped by three parameters, α, λ and β, where α controls the minimum proportion of malaria-positive fevers in a population that can be due to malaria, and λ controls the rate of decline with increasing *Pf*PR$_{0\text{-}5}$ until the minimum proportion of fevers, α, is reached. The relationship is scaled by the parameter β. p_{pos} represents the proportion of individuals who are *P. falciparum*-positive (whether febrile or not). The model used a numerical optimisation function to learn the parameters α, λ, and β (plus an additional standard deviation/observational noise parameter, σ) to fit the relationship between malaria-attributable fevers (as a proportion of malaria-positive fevers) and *Pf*PR$_{0\text{-}5}$ in a two-step process: first, all four parameters were optimised using only the DHS Program dataset, allowing the shape of the relationship to be learnt; second, λ and α were fixed to the optimised values identified in the first model fit using the DHS Program dataset, and the model re-fit β and σ, using the remaining datasets. Using this method, the intercept and rate of decline in p_{MAF} was learnt using the DHS Program dataset (i.e. Dataset 2), and then the relationship was re-scaled by the non-DHS Program datasets (i.e. Datasets 1, 3 and 4: the parasite-density literature review dataset, the paired observations of fever and malaria dataset, and PRISM dataset, as detailed above). For direct comparability between datasets, only observations for children under five years of age were included. All modelling described above was completed in in R using the TMB package.[6]

Details of the literature review and PRISM datasets can be found in Additional File 4.

3. Additional factors investigated

Additional factors which may impact the relationship between MAF and *Pf*PR$_{0\text{-}5}$ were also investigated. First, recent declines in *Pf*PR$_{0\text{-}5}$ may affect the relationship due to residual exposure-dependent immunity, so DHS Program data were segregated by the magnitude of declines in *Pf*PR$_{0\text{-}5}$ during the two years preceding the survey (measured at cluster-level for each 5km x 5km pixel) using cartographic model-based geostatistical estimates for 2000-2016[7] and the model was fit to each data subset to investigate differences. Second, the parasite density threshold dataset (used for the secondary model calibration) was based on active case detection studies, meaning that the prompt and effective treatment rate for malaria cases is typically close to 100%.[2] The treatment rate in all the other datasets was far more variable. The potential effect of differing treatment rates on the relationship between MAF and *Pf*PR$_{0\text{-}5}$ was investigated by segregating the DHS Program data by the treatment-seeking rate within each cluster.

4. References

1 Battle KE, Guerra CA, Golding N, *et al.* Global database of matched *Plasmodium falciparum* and *P . vivax* incidence and prevalence records from 1985 – 2013. *Sci Data* 2015; **2**: 150012.

2 Cameron E, Battle KE, Bhatt S, *et al.* Defining the relationship between infection prevalence and clinical incidence of *Plasmodium falciparum malaria. Nat Commun* 2015; **6**. DOI:10.1038/ncomms9170.

3 DHS Program. 2018. http://dhsprogram.com/ (accessed Aug 28, 2018).

4 Malaria Atlas Project. 2017. http://www.map.ox.ac.uk/ (accessed May 25, 2017).

5 Dorsey G, Kamya M, Greenhouse B, *et al.* Data Set: PRISM Cohort Study. 2018. https://clinepidb.org/ce/app/record/dataset/DS_0ad509829e (accessed June 20, 2018).

6 Kristensen K, Nielsen A, Berg CW, Skaug H, Bell B. TMB: Automatic Differentiation and Laplace Approximation. *J Stat Softw* 2016; **70**. DOI:10.18637/jss.v070.i05.

7 Bhatt S, Weiss D, Cameron E, *et al.* The effect of malaria control on *Plasmodium falciparum* in Africa between 2000 and 2015. *Nature* 2015; **526**: 207–11.
