# Supplementary material for: The contribution of non-malarial febrile illness co-infections to Plasmodium falciparum case counts in health facilities in sub-Saharan Africa
Source: Malar J. 2019 Jun 11;18:195. doi: 10.1186/s12936-019-2830-y (PMC6560910; doi:10.1186/s12936-019-2830-y)
Supplement: Supplementary file 5 — Additional file 5. Details of covariates used in MAF–NMFI modelling approach. [file 12936_2019_2830_MOESM5_ESM.pdf]

Additional File 5. Details of covariates used in MAF-NMFI modelling approach

Three model-derived spatiotemporal estimates were utilised as predictor variables for probabilistic attribution of MAF or non-malarial febrile illness (NMFI) for each febrile individual within the household survey dataset. In order to increase computational efficiency whilst retaining use of the large repository of remotely-sensed data and malaria-related health metrics created and/or collated within the Malaria Atlas Project database, three modelled estimates that incorporate these metrics as predictor variables were used. These three predictor variables were: *i)* $Pf\mathrm{PR}_{0\text{-}5}$, *ii)* suitability for fever without a malaria infection in children under 5 years of age, and *iii)* caregivers' propensity to seek treatment for fever of any cause for children less than 5 years of age. All three variables were generated as cartographic predictions, at a 5km x 5km resolution within areas of stable *P. falciparum* transmission across sub-Saharan Africa, and are described in full below.

The $Pf\mathrm{PR}_{0\text{-}5}$ variable was generated by age-transforming estimates of malaria prevalence via microscopy diagnosis in children 2-10 years of age in Africa, as presented by Bhatt and colleagues,[7] using the 'agestand' package in the R programming language, based on the age-standardisation procedure for *Pf*PR described by Smith *et al.*[1] These estimates were then standardised to RDT-based prevalence estimates of malaria using the methods described by Mappin *et al.* [2]

The remaining two covariates were generated using a boosted regression tree (BRT) approach, and are henceforth referred to as "$\mathrm{BRT_{bg}}$" for suitability for fever without a malaria infection (a "background" fever, hence $\mathrm{BRT_{bg}}$), and "$\mathrm{BRT_{treat}}$" for propensity for all-cause fever treatment-seeking. The approach used is an adapted version of a methodology outlined previously.[3] To generate the two BRT variables, a training dataset was constructed, comprised of the household survey data points described above aggregated into 5km x 5km groups (to match the resolution of the predictor variables), including only clusters with more than ten children interviewed (to remove outliers due to small sample sizes), resulting in a training dataset of 5,262 clusters. For the $\mathrm{BRT_{bg}}$ model, the response variable was the empirical logit of the prevalence of fever without an RDT-patent malaria infection. For the $\mathrm{BRT_{treat}}$ model, the response was the empirical logit of the treatment-seeking rate amongst febrile individuals within each cluster. For both of these models, a suite of 157 environmental and socio-demographic covariates were initially assembled. These covariates, for the area of stable *P. falciparum* transmission in Africa, included 5km x 5km resolution remotely-sensed covariates (and derived products of remotely sensed variables),[4] and annual national-level demographic and poverty indicators supplied by the World Bank.[5] The World Bank data were incomplete for a number of country years, and were gap-filled using a Gaussian process regression approach. The temporal resolution of these datasets was either monthly, yearly, or stationary across the study period. Full details of the variables used and each variable's contribution to each final BRT model are given below.

For both the $\mathrm{BRT_{bg}}$ and $\mathrm{BRT_{treat}}$ covariates, a BRT model using the response dataset and the 157 covariate layers was constructed in the 'dismo' package in R developed by Elith *et al.*[6] The BRT split response data repeatedly by covariates, and the covariates that maximised the homogeneity of each of the two partitioned response datasets were selected more frequently in subsequent steps. The regression tree models were then "boosted", where models were combined to reduce overall predictive deviance. Both models were parameterised using a tree complexity of 5, a learning rate of 0·01, and the number of trees was determined by the optimal solution found by the 'gbm.step' function. Spatial predictions of $\mathrm{BRT_{bg}}$ and $\mathrm{BRT_{treat}}$ were generated across stable *P. falciparum* transmission zones in sub-Saharan Africa on a monthly basis from January 2006 to December 2016. Any gaps in these modelled predictions, caused by areas of missing data in the remotely sensed covariates, were filled with the average value from the nine surrounding cells.

References

1 Smith DL, Guerra CA, Snow RW, Hay SI. Standardizing estimates of the *Plasmodium falciparum* parasite rate. *Malar J* 2007; **6**. DOI:10.1186/1475-2875-6-131.

2 Mappin B, Cameron E, Dalrymple U, *et al.* Standardizing *Plasmodium falciparum* infection prevalence measured via microscopy versus rapid diagnostic test. *Malar J* 2015; **14**. DOI:10.1186/s12936-015-0984-9.

3 Dalrymple U, Cameron E, Bhatt S, Weiss DJ, Gupta S, Gething PW. Quantifying the contribution of *Plasmodium falciparum* malaria to febrile illness amongst African children. *Elife* 2017; **6**: e29198.

4 Weiss DJ, Mappin B, Dalrymple U, *et al.* Re-examining environmental correlates of *Plasmodium falciparum* malaria endemicity: a data-intensive variable selection approach. *Malar J* 2015; **14**. DOI:10.1186/s12936-015-0574-x.

5 World Bank. World Bank Open Data. 2017. http://data.worldbank.org/ (accessed Aug 22, 2017).

6 Elith J, Leathwick JR, Hastie T. A working guide to boosted regression trees. *J Anim Ecol* 2008; **77**: 802–13.

Additional File 5. Predictor variables for BRT model for BRT[bg] listed by contribution to the final model. EVI: Enhanced Vegetation Index; GUF: Global Urban Footprint; LST: Land Surface Temperature; PET - potential evapotranspiration; TCB - Tassled Cap Brightness; TCW - Tassled Cap Wetness; TMI - Topographic Moisture Index; TSI - Temperature Suitability Index; WB - World Bank Variable."Product" definitions for EVI, LST, TSI, TCW and TCB: Monthly - average value over study month; All-years synoptic - mean EVI for all years 2000-2015; Delta dynamic - difference between monthly value and synoptic product; Monthly synoptic - mean of study month across all years; Delta monthly - difference between study month and monthly synoptic product; Delta annual - difference between study month and study year (e.g. July 2008 - Mean 2008). "Lag" defines the number of months that the covariate dataset lags behind the month of each household survey dataset point (e.g. if a household survey data point is recorded in July 2008, a covariate with a lag of 2 months will be taken from May 2008).

Variable name	Contribution to final model	Variable Type	Product type	Spatial summary	Temporal resolution	Monthly lag
primary_school	7.203	Proportion of children in primary school (WB National)	-	N/A	Yearly	N/A
pregnant	5.880	Percentage of pregnant women who receive prenatal care (WB National)	-	N/A	Yearly	N/A
DPT	4.373	DPT immunisation coverage (WB annual)	-	N/A	Yearly	N/A
public_exp	2.656	Public expenditure on health system (WB national)	-	N/A	Yearly	N/A
GDP_grow	2.604	Annual GDP growth (WB national)	-	N/A	Yearly	N/A
prec57p2	1.704	Precipitation	-	N/A	Stationary	N/A
prec57p3	1.618	Precipitation	-	N/A	Stationary	N/A
LST_night_delta_dynamic_mean_1	1.252	LST night	Delta dynamic	Mean	Fully dynamic	1
EVI_delta_annual_min_0	1.237	EVI	Delta annual	Minimum	Delta annual	0
LST_night_delta_monthly_range_4	1.102	LST night	Delta monthly	Range	Fully dynamic	4
EVI_delta_monthly_stdev_4	1.002	EVI	Delta monthly	Standard deviation	Fully dynamic	4
LST_delta_delta_annual_mean_0	1.002	LST delta	Delta annual	Mean	Fully dynamic	0
EVI_delta_dynamic_mean_1	0.977	EVI	Delta dynamic	Mean	Fully dynamic	1
LST_day_delta_annual_max_1	0.972	LST day	Delta annual	Maximum	Fully dynamic	1
TSI.monthly.mean.synoptic.stdev_2	0.970	TSI	Monthly synoptic	Mean	Month only	2
prec57a0	0.924	Precipitation	-	N/A	Stationary	N/A
LST_day.monthly.range.synoptic.stdev_2	0.893	LST day	Monthly synoptic	Standard deviation	Month only	2
LST_day_delta_dynamic_range_2	0.892	LST day	Delta dynamic	Range	Fully dynamic	2
LST_delta.monthly.mean.synoptic.stdev_0	0.875	LST delta	Monthly synoptic	Mean	Month only	0
LST_day_delta_monthly_min_1	0.870	LST day	Delta monthly	Minimum	Fully dynamic	1
TSI_delta_monthly_max_0	0.853	TSI	Delta monthly	Maximum	Fully dynamic	0
LST_night_delta_monthly_min_0	0.838	LST night	Delta monthly	Minimum	Fully dynamic	0
prec57p1	0.822	Precipitation	-	N/A	Stationary	N/A
EVI.monthly.min.synoptic.stdev_4	0.821	EVI	Monthly synoptic	Minimum	Month only	4
TSI.monthly.range.synoptic.mean_3	0.811	TSI	Monthly synoptic	Range	Month only	3

Additional File 5 (Continued)

Variable name	Contribution to final model	Variable Type	Product type	Spatial summary	Temporal resolution	Monthly lag
TSI_delta_monthly_min_4	0.774	TSI	Delta monthly	Minimum	Fully dynamic	4
EVI_delta_monthly_min_1	0.757	EVI	Delta monthly	Minimum	Fully dynamic	1
TCB_delta_annual_stdev_4	0.755	TCB	Delta annual	Standard deviation	Fully dynamic	4
TCB_delta_annual_max_3	0.749	TCB	Delta annual	Maximum	Fully dynamic	3
Africa_TMI_90m.max	0.748	TMI	-	Maximum	Stationary	N/A
accessibility_50k_5km.stdev	0.737	Accessibility	-	Standard deviation	Stationary	N/A
fraction.class.14	0.735	Land cover class fraction	-	N/A	Yearly	N/A
TSI.monthly.mean.synoptic.stdev_3	0.732	TSI	Monthly synoptic	Mean	Month only	3
TCB_delta_monthly_mean_1	0.689	TCB	Delta monthly	Mean	Fully dynamic	1
LST_night_delta_dynamic_min_0	0.678	LST night	Delta dynamic	Minimum	Fully dynamic	0
LST_delta_delta_monthly_mean_0	0.672	LST delta	Delta monthly	Mean	Fully dynamic	0
TCB.monthly.mean.synoptic.stdev_4	0.663	TCB	Monthly synoptic	Mean	Month only	4
LST_delta.monthly.mean.synoptic.stdev_1	0.643	LST delta	Monthly synoptic	Mean	Month only	1
EVI.monthly.range.synoptic.stdev_2	0.640	EVI	Monthly synoptic	Range	Month only	2
LST_night_delta_dynamic_mean_3	0.633	LST night	Delta dynamic	Mean	Fully dynamic	3
EVI_delta_monthly_min_0	0.615	EVI	Delta monthly	Minimum	Fully dynamic	0
TCW_delta_monthly_mean_1	0.606	TCW	Delta monthly	Mean	Fully dynamic	1
LST_day_delta_monthly_stdev_4	0.599	LST day	Delta monthly	Standard deviation	Fully dynamic	4
Africa_TMI_90m.min	0.592	TMI	-	Minimum	Stationary	N/A
LST_night.monthly.stdev.synoptic.stdev_3	0.576	LST night	Monthly synoptic	Standard deviation	Month only	3
EVI_delta_monthly_range_2	0.557	EVI	Delta monthly	Range	Fully dynamic	2
LST_night.monthly.min.synoptic.stdev_2	0.552	LST night	Monthly synoptic	Minimum	Month only	2
fraction.class.9	0.545	Land cover class fraction	-	N/A	Yearly	N/A
TSI_delta_annual_min_4	0.543	TSI	Delta annual	Minimum	Fully dynamic	4
LST_night.monthly.range.synoptic.stdev_1	0.542	LST night	Monthly synoptic	Range	Month only	1
TSI_delta_monthly_max_2	0.540	TSI	Delta monthly	Maximum	Fully dynamic	2
TSI.monthly.min.synoptic.stdev_4	0.539	TSI	Monthly synoptic	Minimum	Month only	4
TCB_delta_annual_stdev_2	0.538	TCB	Delta annual	Standard deviation	Fully dynamic	2
LST_night.monthly.mean.synoptic.stdev_0	0.516	LST night	Monthly synoptic	Mean	Month only	0
LST_delta.monthly.max.synoptic.mean_2	0.510	LST delta	Monthly synoptic	Maximum	Month only	2
prec57a3	0.508	Precipitation	-	N/A	Stationary	N/A

Additional File 5 (Continued)

Variable name	Contribution to final model	Variable Type	Product type	Spatial summary	Temporal resolution	Monthly lag
TSI_delta_monthly_stdev_4	0.507	TSI	Delta monthly	Standard deviation	Fully dynamic	4
TCB_monthly_min_1	0.501	TCB	Monthly	Minimum	Fully dynamic	1
LST_night_delta_dynamic_max_2	0.498	LST night	Delta dynamic	Maximum	Fully dynamic	2
LST_day.monthly.range.synoptic.stdev_1	0.497	LST day	Monthly synoptic	Standard deviation	Month only	1
TSI.monthly.range.synoptic.stdev_2	0.493	TSI	Monthly synoptic	Standard deviation	Month only	2
LST_night_delta_monthly_max_3	0.488	LST night	Delta monthly	Maximum	Fully dynamic	3
LST_night.monthly.stdev.synoptic.stdev_0	0.487	LST night	Monthly synoptic	Standard deviation	Month only	0
LST_night_delta_monthly_mean_4	0.481	LST night	Delta monthly	Mean	Fully dynamic	4
LST_night_delta_monthly_max_2	0.478	LST night	Delta monthly	Maximum	Fully dynamic	2
LST_night_delta_annual_max_2	0.477	LST night	Delta annual	Maximum	Fully dynamic	2
TSI_delta_monthly_range_3	0.474	TSI	Delta monthly	Range	Fully dynamic	3
TCB_delta_annual_range_1	0.454	TCB	Delta annual	Range	Fully dynamic	1
LST_night.monthly.stdev.synoptic.stdev_2	0.449	LST night	Monthly synoptic	Standard deviation	Month only	2
LST_day.monthly.stdev.synoptic.stdev_3	0.444	LST day	Monthly synoptic	Standard deviation	Month only	3
TSI_delta_monthly_mean_1	0.444	TSI	Delta monthly	Mean	Fully dynamic	1
LST_day_delta_monthly_max_4	0.439	LST day	Delta monthly	Maximum	Fully dynamic	4
TCW_delta_annual_stdev_0	0.436	TCW	Delta annual	Standard deviation	Fully dynamic	0
LST_day_delta_dynamic_stdev_0	0.435	LST day	Delta dynamic	Standard deviation	Fully dynamic	0
EVI.monthly.mean.synoptic.stdev_2	0.435	EVI	Monthly synoptic	Standard deviation	Month only	2
EVI_all_years_synoptic_min	0.431	EVI	All-years synoptic	Minimum	Stationary	N/A
EVI.monthly.stdev.synoptic.stdev_4	0.429	EVI	Monthly synoptic	Standard deviation	Month only	4
EVI_delta_dynamic_min_4	0.427	EVI	Delta dynamic	Minimum	Fully dynamic	4
EVI_delta_monthly_stdev_0	0.423	EVI	Delta monthly	Standard deviation	Fully dynamic	0
LST_night.monthly.mean.synoptic.stdev_1	0.415	LST night	Monthly synoptic	Mean	Month only	1
LST_night.monthly.max.synoptic.stdev_4	0.413	LST night	Monthly synoptic	Maximum	Month only	4
TCW_delta_monthly_mean_4	0.413	TCW	Delta monthly	Mean	Fully dynamic	4
population_covariates	0.412	Population density	-	N/A	Yearly	N/A
LST_night_delta_dynamic_stdev_0	0.410	LST night	Delta dynamic	Standard deviation	Fully dynamic	0
Africa_FA_90m.mean	0.409	Elevation (flow accumulation)	-	Mean	Stationary	N/A
TCW_monthly_stdev_3	0.408	TCW	Monthly	Standard deviation	Fully dynamic	3
TSI.monthly.max.synoptic.stdev_4	0.407	TSI	Monthly synoptic	Maximum	Month only	4

Additional File 5 (Continued)

Variable name	Contribution to final model	Variable Type	Product type	Spatial summary	Temporal resolution	Monthly lag
EVI_delta_monthly_min_2	0.403	EVI	Delta monthly	Minimum	Fully dynamic	2
LST_day_delta_monthly_range_1	0.403	LST day	Delta monthly	Range	Fully dynamic	1
EVI_delta_monthly_stdev_1	0.400	EVI	Delta monthly	Standard deviation	Fully dynamic	1
adjacencies.class.8	0.398	Land cover adjacencies	-	N/A	Yearly	N/A
LST_day.monthly.min.synoptic.stdev_2	0.395	LST day	Monthly synoptic	Minimum	Month only	2
TCW_delta_monthly_mean_0	0.394	TCW	Delta monthly	Mean	Fully dynamic	0
TSI_delta_monthly_max_4	0.389	TSI	Delta monthly	Maximum	Fully dynamic	4
PET_1950.2000_5km.stdev	0.388	PET	-	Standard deviation	Stationary	N/A
viirs_nighttime_5km.mean	0.385	Nighttime lights	-	Mean	Stationary	N/A
EVI.monthly.range.synoptic.stdev_0	0.380	EVI	Monthly synoptic	Range	Month only	0
LST_day_delta_annual_stdev_3	0.379	LST day	Delta annual	Standard deviation	Fully dynamic	3
Global_Urban_Footprint_5km_PropUrban	0.379	Proportion urban in cell (GUF)	-	N/A	Stationary	N/A
LST_night.monthly.range.synoptic.mean_1	0.375	LST night	Monthly synoptic	Range	Month only	1
fraction.class.2	0.369	Land cover class fraction	-	N/A	Yearly	N/A
TCW_delta_annual_stdev_3	0.358	TCW	Delta annual	Standard deviation	Fully dynamic	3
LST_night_delta_annual_range_1	0.356	LST night	Delta annual	Range	Fully dynamic	1
population_density_5km.min	0.355	Population density	-	Minimum	Stationary	N/A
LST_day_delta_monthly_min_3	0.350	LST day	Delta monthly	Minimum	Fully dynamic	3
LST_day_delta_monthly_range_0	0.348	LST day	Delta monthly	Range	Fully dynamic	0
EVI_delta_dynamic_stdev_1	0.346	EVI	Delta dynamic	Standard deviation	Fully dynamic	1
LST_day.monthly.stdev.synoptic.stdev_4	0.341	LST day	Monthly synoptic	Standard deviation	Month only	4
TCW_delta_monthly_mean_2	0.341	TCW	Delta monthly	Mean	Fully dynamic	2
TSI_delta_monthly_stdev_2	0.340	TSI	Delta monthly	Standard deviation	Fully dynamic	2
TCB_delta_monthly_mean_0	0.339	TCB	Delta monthly	Mean	Fully dynamic	0
TCB_delta_dynamic_mean_4	0.337	TCB	Delta monthly	Mean	Fully dynamic	4
prec57a1	0.335	Precipitation	-	N/A	Stationary	N/A
TCW_delta_monthly_mean_3	0.332	TCW	Delta monthly	Mean	Fully dynamic	3
TCB_delta_monthly_mean_4	0.330	TCB	Delta monthly	Mean	Fully dynamic	4
TSI.monthly.min.synoptic.stdev_0	0.329	TSI	Monthly synoptic	Minimum	Month only	0
LST_day.monthly.mean.synoptic.stdev_3	0.325	LST day	Monthly synoptic	Mean	Month only	3
EVI_delta_dynamic_range_0	0.320	EVI	Delta dynamic	Range	Fully dynamic	0

Additional File 5 (Continued)

Variable name	Contribution to final model	Variable Type	Product type	Spatial summary	Temporal resolution	Monthly lag
TCB_monthly_max_4	0.320	TCB	Monthly	Maximum	Fully dynamic	4
TSI_delta_monthly_max_3	0.320	TSI	Delta monthly	Maximum	Fully dynamic	3
LST_night_delta_monthly_max_1	0.318	LST night	Delta monthly	Maximum	Fully dynamic	1
LST_delta_delta_monthly_mean_2	0.316	LST delta	Delta monthly	Mean	Fully dynamic	2
TCB_delta_monthly_mean_2	0.312	TCB	Delta monthly	Mean	Fully dynamic	2
TSI_delta_monthly_min_2	0.312	TSI	Delta monthly	Minimum	Fully dynamic	2
LST_delta_all_years_synoptic_max	0.311	LST delta	All-years synoptic	Maximum	Stationary	N/A
LST_night.monthly.stdev.synoptic.mean_4	0.311	LST night	Monthly synoptic	Standard deviation	Month only	4
LST_night_delta_dynamic_max_4	0.310	LST night	Delta dynamic	Maximum	Fully dynamic	4
EVI_delta_monthly_mean_3	0.307	EVI	Delta monthly	Mean	Fully dynamic	3
LST_night_delta_monthly_range_0	0.295	LST night	Delta monthly	Range	Fully dynamic	0
EVI_delta_monthly_range_3	0.293	EVI	Delta monthly	Range	Fully dynamic	3
LST_night_delta_annual_stdev_3	0.281	LST night	Delta annual	Standard deviation	Fully dynamic	3
LST_night_delta_dynamic_stdev_4	0.281	LST night	Delta dynamic	Standard deviation	Fully dynamic	4
TSI_monthly_stdev_1	0.277	TSI	Monthly	Standard deviation	Fully dynamic	1
EVI_delta_dynamic_range_4	0.276	EVI	Delta dynamic	Range	Fully dynamic	4
EVI.monthly.mean.synoptic.stdev_0	0.275	EVI	Monthly synoptic	Mean	Month only	0
LST_day_delta_monthly_stdev_3	0.269	LST day	Delta monthly	Standard deviation	Fully dynamic	3
fraction.class.5	0.268	Land cover class fraction	-	N/A	Yearly	N/A
prec57a2	0.260	Precipitation	-	N/A	Stationary	N/A
LST_night_delta_monthly_stdev_2	0.256	LST night	Delta monthly	Standard deviation	Fully dynamic	2
TCW_delta_annual_stdev_1	0.253	TCW	Delta annual	Standard deviation	Fully dynamic	1
TCB_delta_annual_range_3	0.247	TCB	Delta annual	Range	Fully dynamic	3
TCW_delta_annual_stdev_4	0.247	TCW	Delta annual	Standard deviation	Fully dynamic	4
EVI_delta_monthly_max_2	0.244	EVI	Delta monthly	Maximum	Fully dynamic	2
TSI.monthly.mean.synoptic.stdev_1	0.241	TSI	Monthly synoptic	Mean	Month only	1
TSI_delta_monthly_min_3	0.239	TSI	Delta monthly	Minimum	Fully dynamic	3
LST_night_delta_annual_stdev_2	0.238	LST night	Delta annual	Standard deviation	Fully dynamic	2
LST_day.monthly.range.synoptic.mean_4	0.231	LST day	Monthly synoptic	Range	Month only	4
EVI_all_years_synoptic_range	0.229	EVI	All-years synoptic	Range	Stationary	N/A
LST_day_delta_annual_range_4	0.226	LST day	Delta annual	Range	Fully dynamic	4

Additional File 5 (Continued)

Variable name	Contribution to final model	Variable Type	Product type	Spatial summary	Temporal resolution	Monthly lag
LST_day.monthly.range.synoptic.stdev_0	0.226	LST day	Monthly synoptic	Range	Month only	0
TSI_delta_monthly_range_0	0.226	TSI	Delta monthly	Range	Fully dynamic	0
LST_delta_delta_annual_max_2	0.223	LST delta	Delta annual	Maximum	Fully dynamic	2
TCW_delta_annual_stdev_2	0.223	TCW	Delta annual	Standard deviation	Fully dynamic	2
accessibility_ESRI_5km.range	0.222	Accessibility	-	Range	Stationary	N/A
Africa_TMI_90m.stdev	0.214	TMI	-	Standard deviation	Stationary	N/A
EVI_delta_annual_stdev_2	0.206	EVI	Delta annual	Standard deviation	Delta annual	2
fraction.class.7	0.206	Land cover class fraction	-	N/A	Yearly	N/A
TCW_delta_dynamic_mean_2	0.200	TCW	Delta dynamic	Mean	Fully dynamic	2
fraction.class.12	0.199	Land cover class fraction	-	N/A	Yearly	N/A
LST_night.monthly.min.synoptic.stdev_3	0.197	LST night	Monthly synoptic	Minimum	Month only	3
TSI_monthly_stdev_2	0.188	TSI	Monthly	Standard deviation	Fully dynamic	2
EVI_delta_monthly_mean_4	0.171	EVI	Delta monthly	Mean	Fully dynamic	4
LST_night.monthly.stdev.synoptic.stdev_4	0.164	LST night	Monthly synoptic	Standard deviation	Month only	4
EVI_delta_dynamic_stdev_3	0.163	EVI	Delta dynamic	Standard deviation	Fully dynamic	3
TSI.monthly.range.synoptic.stdev_1	0.156	TSI	Monthly synoptic	Standard deviation	Month only	1
population_density_5km.stdev	0.145	Population density	-	Standard deviation	Stationary	N/A
DMSP_F18_5km.max	0.113	Nighttime lights	-	Maximum	Stationary	N/A
fraction.class.10	0.069	Land cover class fraction	-	N/A	Yearly	N/A
fraction.class.11	0.065	Land cover class fraction	-	N/A	Yearly	N/A
majclass_covariates	0.064	Land cover majority class	-	N/A	Yearly	N/A
Africa_slope_90m.min	0.026	Elevation (slope angle)	-	Minimum	Stationary	N/A
TCW_delta_annual_max_2	0.005	TCW	Delta annual	Maximum	Fully dynamic	2
Global_Urban_Footprint_5km_AnyUrban	0.000	Any urban in cell (GUF)	-	N/A	Stationary	N/A
Global_Urban_Footprint_5km_SignificantUrb	0.000	Significant urban in cell (GUF)	-	N/A	Stationary	N/A
adjacencies.class.16	0.000	Land cover adjacencies	-	N/A	Yearly	N/A

Additional File 5. Predictor variables for BRT model for BRT[treat] listed by contribution to the final model. EVI: Enhanced Vegetation Index; GUF: Global Urban Footprint; LST: Land Surface Temperature; PET - potential evapotranspiration; TCB - Tassled Cap Brightness; TCW - Tassled Cap Wetness; TMI - Topographic Moisture Index; TSI - Temperature Suitability Index; WB - World Bank Variable.“Product” definitions for EVI, LST, TSI, TCW and TCB: Monthly - average value over study month; All-years synoptic - mean EVI for all years 2000-2015; Delta dynamic - difference between monthly value and synoptic product; Monthly synoptic - mean of study month across all years; Delta monthly - difference between study month and monthly synoptic product; Delta annual - difference between study month and study year (e.g. July 2008 - Mean 2008). “Lag” defines the number of months that the covariate dataset lags behind the month of each household survey dataset point (e.g. if a household survey data point is recorded in July 2008, a covariate with a lag of 2 months will be taken from May 2008).

**Variable name**	**Contribution to final model**	**Variable Type**	**Product type**	**Spatial summary**	**Temporal resolution**	**Monthly lag**
GDP_grow	14.157	Annual GDP growth (WB national)	-	N/A	Yearly	N/A
public_exp	7.680	Public expenditure on health system (WB national)	-	N/A	Yearly	N/A
primary_school	3.972	Proportion of children in primary school (WB National)	-	N/A	Yearly	N/A
prec57p3	1.820	Precipitation	-	N/A	Stationary	N/A
LST_delta_delta_annual_max_2	1.753	LST delta	Delta annual	Maximum	Fully dynamic	2
prec57a0	1.701	Precipitation	-	N/A	Stationary	N/A
prec57a1	1.582	Precipitation	-	N/A	Stationary	N/A
LST_night_delta_monthly_max_3	1.418	LST night	Delta monthly	Maximum	Fully dynamic	3
fraction.class.10	1.385	Land cover class fraction	-	N/A	Yearly	N/A
pregnant	1.360	Percentage of pregnant women who receive prenatal care (WB National)	-	N/A	Yearly	N/A
LST_night_delta_dynamic_max_4	1.252	LST night	Delta dynamic	Maximum	Fully dynamic	4
population_covariates	1.101	Population density	-	N/A	Yearly	N/A
LST_night.monthly.mean.synoptic.stdev_1	1.081	LST night	Monthly synoptic	Mean	Month only	1
TSI_delta_monthly_min_3	1.055	TSI	Delta monthly	Minimum	Fully dynamic	3
PET_1950.2000_5km.stdev	1.049	PET	-	Standard deviation	Stationary	N/A
TSI_delta_monthly_max_3	1.007	TSI	Delta monthly	Maximum	Fully dynamic	3
LST_night_delta_monthly_max_1	0.998	LST night	Delta monthly	Maximum	Fully dynamic	1
EVI_delta_dynamic_min_4	0.965	EVI	Delta dynamic	Minimum	Fully dynamic	4
LST_day_delta_monthly_max_4	0.956	LST day	Delta monthly	Maximum	Fully dynamic	4
LST_delta.monthly.mean.synoptic.stdev_1	0.949	LST delta	Monthly synoptic	Mean	Month only	1

Additional File 5 (Continued)

Variable name	Contribution to final model	Variable Type	Product type	Spatial summary	Temporal resolution	Monthly lag
LST_night.monthly.stdev.synoptic.stdev_0	0.901	LST night	Monthly synoptic	Standard deviation	Month only	0
LST_day_delta_dynamic_stdev_0	0.884	LST day	Delta dynamic	Standard deviation	Fully dynamic	0
TSI_delta_monthly_max_4	0.845	TSI	Delta monthly	Maximum	Fully dynamic	4
LST_day.monthly.range.synoptic.stdev_0	0.814	LST day	Monthly synoptic	Range	Month only	0
LST_night.monthly.stdev.synoptic.stdev_3	0.795	LST night	Monthly synoptic	Standard deviation	Month only	3
LST_delta_delta_monthly_mean_0	0.767	LST delta	Delta monthly	Mean	Fully dynamic	0
TCB_delta_dynamic_mean_4	0.758	TCB	Delta monthly	Mean	Fully dynamic	4
EVI_delta_monthly_mean_3	0.737	EVI	Delta monthly	Mean	Fully dynamic	3
population_density_5km.min	0.731	Population density	-	Minimum	Stationary	N/A
TSI_delta_monthly_stdev_4	0.703	TSI	Delta monthly	Standard deviation	Fully dynamic	4
Global_Urban_Footprint_5km_PropUrban	0.700	Proportion urban in cell (GUF)	-	N/A	Stationary	N/A
EVI.monthly.range.synoptic.stdev_2	0.699	EVI	Monthly synoptic	Range	Month only	2
LST_night_delta_annual_stdev_2	0.674	LST night	Delta annual	Standard deviation	Fully dynamic	2
TCB_monthly_max_4	0.663	TCB	Monthly	Maximum	Fully dynamic	4
LST_delta_all_years_synoptic_max	0.655	LST delta	All-years synoptic	Maximum	Stationary	N/A
prec57a3	0.634	Precipitation	-	N/A	Stationary	N/A
TCW_delta_monthly_mean_4	0.634	TCW	Delta monthly	Mean	Fully dynamic	4
EVI_delta_monthly_stdev_1	0.629	EVI	Delta monthly	Standard deviation	Fully dynamic	1
LST_night.monthly.stdev.synoptic.stdev_4	0.626	LST night	Monthly synoptic	Standard deviation	Month only	4
LST_delta.monthly.mean.synoptic.stdev_0	0.618	LST delta	Monthly synoptic	Mean	Month only	0
EVI_delta_monthly_mean_4	0.614	EVI	Delta monthly	Mean	Fully dynamic	4
LST_night_delta_monthly_max_2	0.609	LST night	Delta monthly	Maximum	Fully dynamic	2
EVI_delta_dynamic_mean_1	0.598	EVI	Delta dynamic	Mean	Fully dynamic	1
LST_day_delta_monthly_range_0	0.587	LST day	Delta monthly	Range	Fully dynamic	0
LST_night_delta_monthly_mean_4	0.574	LST night	Delta monthly	Mean	Fully dynamic	4
TCW_delta_annual_stdev_0	0.559	TCW	Delta annual	Standard deviation	Fully dynamic	0
TSI_delta_monthly_max_0	0.532	TSI	Delta monthly	Maximum	Fully dynamic	0
LST_night_delta_annual_stdev_3	0.514	LST night	Delta annual	Standard deviation	Fully dynamic	3
TSI.monthly.mean.synoptic.stdev_3	0.506	TSI	Monthly synoptic	Mean	Month only	3
LST_day.monthly.range.synoptic.stdev_1	0.500	LST day	Monthly synoptic	Standard deviation	Month only	1
TSI_delta_monthly_min_4	0.495	TSI	Delta monthly	Minimum	Fully dynamic	4

Additional File 5 (Continued)						
**Variable name**	**Contribution to final model**	**Variable Type**	**Product type**	**Spatial summary**	**Temporal resolution**	**Monthly lag**
TCB_delta_monthly_mean_0	0.477	TCB	Delta monthly	Mean	Fully dynamic	0
viirs_nighttime_5km.mean	0.465	Nighttime lights	-	Mean	Stationary	N/A
TSI_delta_annual_min_4	0.459	TSI	Delta annual	Minimum	Fully dynamic	4
prec57p1	0.456	Precipitation	-	N/A	Stationary	N/A
LST_day_delta_monthly_range_1	0.455	LST day	Delta monthly	Range	Fully dynamic	1
TSI.monthly.mean.synoptic.stdev_2	0.441	TSI	Monthly synoptic	Mean	Month only	2
fraction.class.12	0.437	Land cover class fraction	-	N/A	Yearly	N/A
LST_day.monthly.stdev.synoptic.stdev_3	0.419	LST day	Monthly synoptic	Standard deviation	Month only	3
TCB_delta_annual_range_3	0.406	TCB	Delta annual	Range	Fully dynamic	3
LST_delta.monthly.max.synoptic.mean_2	0.404	LST delta	Monthly synoptic	Maximum	Month only	2
LST_day_delta_annual_stdev_3	0.401	LST day	Delta annual	Standard deviation	Fully dynamic	3
TCW_delta_monthly_mean_2	0.395	TCW	Delta monthly	Mean	Fully dynamic	2
TCB_delta_annual_stdev_2	0.395	TCB	Delta annual	Standard deviation	Fully dynamic	2
TCB_delta_annual_stdev_4	0.394	TCB	Delta annual	Standard deviation	Fully dynamic	4
TSI_delta_monthly_range_3	0.390	TSI	Delta monthly	Range	Fully dynamic	3
prec57p2	0.386	Precipitation	-	N/A	Stationary	N/A
EVI.monthly.mean.synoptic.stdev_2	0.385	EVI	Monthly synoptic	Standard deviation	Month only	2
LST_day_delta_monthly_stdev_4	0.381	LST day	Delta monthly	Standard deviation	Fully dynamic	4
LST_night_delta_dynamic_mean_1	0.376	LST night	Delta dynamic	Mean	Fully dynamic	1
EVI_delta_annual_min_0	0.376	EVI	Delta annual	Minimum	Delta annual	0
TCB_delta_monthly_mean_4	0.373	TCB	Delta monthly	Mean	Fully dynamic	4
LST_night_delta_monthly_stdev_2	0.372	LST night	Delta monthly	Standard deviation	Fully dynamic	2
EVI_delta_dynamic_range_4	0.371	EVI	Delta dynamic	Range	Fully dynamic	4
TCW_delta_dynamic_mean_2	0.365	TCW	Delta dynamic	Mean	Fully dynamic	2
LST_night.monthly.min.synoptic.stdev_2	0.364	LST night	Monthly synoptic	Minimum	Month only	2
TSI_delta_monthly_max_2	0.355	TSI	Delta monthly	Maximum	Fully dynamic	2
EVI_all_years_synoptic_range	0.354	EVI	All-years synoptic	Range	Stationary	N/A
LST_day.monthly.min.synoptic.stdev_2	0.353	LST day	Monthly synoptic	Minimum	Month only	2
DPT	0.344	DPT immunisation coverage (WB annual)	-	N/A	Yearly	N/A
LST_night_delta_dynamic_min_0	0.343	LST night	Delta dynamic	Minimum	Fully dynamic	0
LST_night_delta_monthly_min_0	0.340	LST night	Delta monthly	Minimum	Fully dynamic	0

Additional File 5 (Continued)

Variable name	Contribution to final model	Variable Type	Product type	Spatial summary	Temporal resolution	Monthly lag
TSI_delta_monthly_stdev_2	0.339	TSI	Delta monthly	Standard deviation	Fully dynamic	2
EVI_delta_monthly_range_3	0.337	EVI	Delta monthly	Range	Fully dynamic	3
EVI_delta_monthly_range_2	0.336	EVI	Delta monthly	Range	Fully dynamic	2
TSI.monthly.mean.synoptic.stdev_1	0.332	TSI	Monthly synoptic	Mean	Month only	1
TCW_delta_annual_stdev_4	0.332	TCW	Delta annual	Standard deviation	Fully dynamic	4
LST_day_delta_annual_max_1	0.328	LST day	Delta annual	Maximum	Fully dynamic	1
LST_day_delta_monthly_min_3	0.328	LST day	Delta monthly	Minimum	Fully dynamic	3
LST_night.monthly.range.synoptic.mean_1	0.325	LST night	Monthly synoptic	Range	Month only	1
TSI_delta_monthly_min_2	0.321	TSI	Delta monthly	Minimum	Fully dynamic	2
LST_night_delta_annual_max_2	0.320	LST night	Delta annual	Maximum	Fully dynamic	2
EVI_delta_monthly_min_1	0.319	EVI	Delta monthly	Minimum	Fully dynamic	1
TCW_delta_annual_stdev_1	0.319	TCW	Delta annual	Standard deviation	Fully dynamic	1
TSI_delta_monthly_range_0	0.317	TSI	Delta monthly	Range	Fully dynamic	0
Africa_TMI_90m.min	0.317	TMI	-	Minimum	Stationary	N/A
LST_day_delta_monthly_stdev_3	0.311	LST day	Delta monthly	Standard deviation	Fully dynamic	3
EVI_all_years_synoptic_min	0.310	EVI	All-years synoptic	Minimum	Stationary	N/A
TCB_monthly_min_1	0.308	TCB	Monthly	Minimum	Fully dynamic	1
LST_night.monthly.max.synoptic.stdev_4	0.304	LST night	Monthly synoptic	Maximum	Month only	4
LST_night.monthly.mean.synoptic.stdev_0	0.303	LST night	Monthly synoptic	Mean	Month only	0
LST_night_delta_dynamic_mean_3	0.287	LST night	Delta dynamic	Mean	Fully dynamic	3
TSI.monthly.max.synoptic.stdev_4	0.286	TSI	Monthly synoptic	Maximum	Month only	4
LST_night_delta_dynamic_stdev_0	0.285	LST night	Delta dynamic	Standard deviation	Fully dynamic	0
LST_night_delta_annual_range_1	0.282	LST night	Delta annual	Range	Fully dynamic	1
LST_night.monthly.stdev.synoptic.stdev_2	0.280	LST night	Monthly synoptic	Standard deviation	Month only	2
EVI_delta_dynamic_stdev_1	0.274	EVI	Delta dynamic	Standard deviation	Fully dynamic	1
EVI_delta_dynamic_range_0	0.263	EVI	Delta dynamic	Range	Fully dynamic	0
TCB_delta_annual_range_1	0.259	TCB	Delta annual	Range	Fully dynamic	1
TSI.monthly.min.synoptic.stdev_0	0.257	TSI	Monthly synoptic	Minimum	Month only	0
TSI.monthly.range.synoptic.mean_3	0.255	TSI	Monthly synoptic	Range	Month only	3
TSI.monthly.range.synoptic.stdev_2	0.255	TSI	Monthly synoptic	Standard deviation	Month only	2
TSI.monthly.range.synoptic.stdev_1	0.252	TSI	Monthly synoptic	Standard deviation	Month only	1
**Additional File 5 (Continued)**						
**Variable name**	**Contribution to final model**	**Variable Type**	**Product type**	**Spatial summary**	**Temporal resolution**	**Monthly lag**

EVI.monthly.mean.synoptic.stdev_0	0.251	EVI	Monthly synoptic	Mean	Month only	0
TCB_delta_monthly_mean_1	0.250	TCB	Delta monthly	Mean	Fully dynamic	1
EVI_delta_monthly_min_0	0.248	EVI	Delta monthly	Minimum	Fully dynamic	0
accessibility_50k_5km.stdev	0.246	Accessibility	-	Standard deviation	Stationary	N/A
LST_day_delta_monthly_min_1	0.246	LST day	Delta monthly	Minimum	Fully dynamic	1
EVI_delta_monthly_max_2	0.246	EVI	Delta monthly	Maximum	Fully dynamic	2
TSI.monthly.min.synoptic.stdev_4	0.240	TSI	Monthly synoptic	Minimum	Month only	4
TCB.monthly.mean.synoptic.stdev_4	0.237	TCB	Monthly synoptic	Mean	Month only	4
EVI.monthly.range.synoptic.stdev_0	0.233	EVI	Monthly synoptic	Range	Month only	0
LST_night_delta_monthly_range_4	0.232	LST night	Delta monthly	Range	Fully dynamic	4
TCW_monthly_stdev_3	0.230	TCW	Monthly	Standard deviation	Fully dynamic	3
TCB_delta_monthly_mean_2	0.228	TCB	Delta monthly	Mean	Fully dynamic	2
DMSP_F18_5km.max	0.228	Nighttime lights	-	Maximum	Stationary	N/A
EVI_delta_dynamic_stdev_3	0.228	EVI	Delta dynamic	Standard deviation	Fully dynamic	3
Africa_FA_90m.mean	0.226	Elevation (flow accumulation)	-	Mean	Stationary	N/A
accessibility_ESRI_5km.range	0.223	Accessibility	-	Range	Stationary	N/A
prec57a2	0.223	Precipitation	-	N/A	Stationary	N/A
EVI_delta_monthly_stdev_0	0.222	EVI	Delta monthly	Standard deviation	Fully dynamic	0
EVI_delta_monthly_min_2	0.221	EVI	Delta monthly	Minimum	Fully dynamic	2
LST_night_delta_monthly_range_0	0.220	LST night	Delta monthly	Range	Fully dynamic	0
EVI_delta_monthly_stdev_4	0.219	EVI	Delta monthly	Standard deviation	Fully dynamic	4
LST_day.monthly.range.synoptic.stdev_2	0.216	LST day	Monthly synoptic	Standard deviation	Month only	2
TCW_delta_monthly_mean_0	0.214	TCW	Delta monthly	Mean	Fully dynamic	0
LST_night_delta_dynamic_max_2	0.214	LST night	Delta dynamic	Maximum	Fully dynamic	2
LST_delta_delta_monthly_mean_2	0.212	LST delta	Delta monthly	Mean	Fully dynamic	2
TSI_delta_monthly_mean_1	0.209	TSI	Delta monthly	Mean	Fully dynamic	1
TCB_delta_annual_max_3	0.207	TCB	Delta annual	Maximum	Fully dynamic	3
fraction.class.14	0.203	Land cover class fraction	-	N/A	Yearly	N/A
EVI_delta_annual_stdev_2	0.196	EVI	Delta annual	Standard deviation	Delta annual	2
LST_night.monthly.min.synoptic.stdev_3	0.195	LST night	Monthly synoptic	Minimum	Month only	3
LST_delta_delta_annual_mean_0	0.194	LST delta	Delta annual	Mean	Fully dynamic	0
**Additional File 5 (Continued)**						
**Variable name**	**Contribution to final model**	**Variable Type**	**Product type**	**Spatial summary**	**Temporal resolution**	**Monthly lag**
LST_day_delta_dynamic_range_2	0.193	LST day	Delta dynamic	Range	Fully dynamic	2
LST_night.monthly.range.synoptic.stdev_1	0.192	LST night	Monthly synoptic	Range	Month only	1

LST_day.monthly.stdev.synoptic.stdev_4	0.192	LST day	Monthly synoptic	Standard deviation	Month only	4
EVI.monthly.min.synoptic.stdev_4	0.188	EVI	Monthly synoptic	Minimum	Month only	4
LST_day_delta_annual_range_4	0.187	LST day	Delta annual	Range	Fully dynamic	4
TCW_delta_annual_stdev_3	0.187	TCW	Delta annual	Standard deviation	Fully dynamic	3
Africa_TMI_90m.stdev	0.184	TMI	-	Standard deviation	Stationary	N/A
LST_day.monthly.mean.synoptic.stdev_3	0.182	LST day	Monthly synoptic	Mean	Month only	3
LST_night_delta_dynamic_stdev_4	0.181	LST night	Delta dynamic	Standard deviation	Fully dynamic	4
EVI.monthly.stdev.synoptic.stdev_4	0.178	EVI	Monthly synoptic	Standard deviation	Month only	4
TSI_monthly_stdev_1	0.178	TSI	Monthly	Standard deviation	Fully dynamic	1
Africa_TMI_90m.max	0.176	TMI	-	Maximum	Stationary	N/A
TCW_delta_monthly_mean_1	0.167	TCW	Delta monthly	Mean	Fully dynamic	1
adjacencies.class.16	0.163	Land cover adjacencies	-	N/A	Yearly	N/A
TCW_delta_monthly_mean_3	0.153	TCW	Delta monthly	Mean	Fully dynamic	3
LST_night.monthly.stdev.synoptic.mean_4	0.149	LST night	Monthly synoptic	Standard deviation	Month only	4
TCW_delta_annual_stdev_2	0.148	TCW	Delta annual	Standard deviation	Fully dynamic	2
TSI_monthly_stdev_2	0.146	TSI	Monthly	Standard deviation	Fully dynamic	2
LST_day.monthly.range.synoptic.mean_4	0.110	LST day	Monthly synoptic	Range	Month only	4
adjacencies.class.8	0.087	Land cover adjacencies	-	N/A	Yearly	N/A
fraction.class.7	0.071	Land cover class fraction	-	N/A	Yearly	N/A
fraction.class.2	0.059	Land cover class fraction	-	N/A	Yearly	N/A
majclass_covariates	0.058	Land cover majority class	-	N/A	Yearly	N/A
population_density_5km.stdev	0.039	Population density	-	Standard deviation	Stationary	N/A
fraction.class.9	0.033	Land cover class fraction	-	N/A	Yearly	N/A
Africa_slope_90m.min	0.029	Elevation (slope angle)	-	Minimum	Stationary	N/A
fraction.class.11	0.025	Land cover class fraction	-	N/A	Yearly	N/A
Global_Urban_Footprint_5km_AnyUrban	0.009	Any urban in cell (GUF)	-	N/A	Stationary	N/A
TCW_delta_annual_max_2	0.008	TCW	Delta annual	Maximum	Fully dynamic	2
fraction.class.5	0.001	Land cover class fraction	-	N/A	Yearly	N/A
Global_Urban_Footprint_5km_SignificantUrb	0.000	Significant urban in cell (GUF)	-	N/A	Stationary	N/A
