# Supplementary material for: The contribution of non-malarial febrile illness co-infections to Plasmodium falciparum case counts in health facilities in sub-Saharan Africa
Source: Malar J. 2019 Jun 11;18:195. doi: 10.1186/s12936-019-2830-y (PMC6560910; doi:10.1186/s12936-019-2830-y)
Supplement: Supplementary file 6 — Additional file 6. Model for individual-level attribution of MAF and NMFI to febrile children within the household survey dataset. [file 12936_2019_2830_MOESM6_ESM.pdf]

Additional File 6. Model for individual-level attribution of MAF and NMFI to febrile children within the household survey dataset

The model utilised in this analysis is a further adaptation of a previously presented model for estimating the prevalence of MAF and NMFI at a 5km x 5km spatial resolution across sub-Saharan Africa from 2006-2014.[10] Here, this model was modified to take into account additional individual-level data on socioeconomic status and treatment-seeking behaviour in the two weeks preceding the survey. This individual-level data can markedly improve the precision of the probabilistic estimations of fever type (i.e. MAF or NMFI), but since it is unavailable as a continuous spatial covariate, population-level estimates cannot be formed by applying the modified model across all pixels within a given country or district. Instead, a mixed estimation strategy is presented in this analysis in which Bayesian posterior samples of predicted fever type were compiled using the design weights of the original survey, as described below.

The individual-level model presented in this analysis assigned a probability of each individual experiencing within the past 14 days a NMFI, a MAF, or both (i.e. a co-symptomatic MAF and NMFI). Conditional on those events a further set of probabilities was computed: probability of treatment-seeking, of receiving a heel-prick diagnosis (used as a proxy for malaria diagnosis at the health clinic), of receiving antimalarials, and of clearing any parasites/antigens early enough to clear an RDT test at time of survey (as defined by the adjustment presented in [1]). Each individual's observed category according to their RDT test result and response to the corresponding survey questions informed their contribution to the likelihood function. The probabilities assigned to parasite positivity status ($p_{\text{pos.}}$), NMFI status (background fever; p_{bg}), and of treatment-seeking under each of the three fever cases—MAF-only ($p_{\text{treat|maf}}$), NMFI-only ($p_{\text{treat|bg}}$), or both ($p_{\text{treat|both}}$)—were estimated from the combination of individual level covariates—mother's education (m_i), child's age (a_i), household wealth (w_i), and bed net use (b_i)—and separate spatial random fields ($f_{\text{pos.}}$, f_{bg}, $f_{\text{treat|maf}}$, $f_{\text{treat|bg}}$, $f_{\text{treat|both}}$). Spatial covariates from high resolution imaging were introduced as described in the previous sub-section via treatment-seeking ($\text{BRT}_{\text{treat}}$) and malaria-negative fever prevalence (BRT_{bg}) predictions from separate BRT models, and malaria prevalence predictions ($\text{MAP}_{\text{pos.}}$) from a published *P. falciparum* prevalence model.[7] The probabilities of each stage of diagnosis and treatment response were assigned survey-level random effects: the probability of receiving a heel-prick test (p_{prick}), the probability of (explicit) 'under-treatment' (not receiving antimalarials despite a positive test result, $p_{\text{undertreat}}$), the probability of (explicit) 'over-treatment' (probability of receiving antimalarials despite a negative test result, $p_{\text{overtreat}}$), and the probability of presumptive treatment (receiving antimalarials without a heel-prick test, $p_{\text{presumptive}}$). Again, the probability of experiencing a MAF given a patent parasite level infection was given by a polynomial function of the background community prevalence to reflect the role of exposure-based immunity.[2-5] For individuals who were RDT-negative at the time of interview but were known to have received antimalarials within the past two weeks, the probability of the individual having been RDT-positive at the time of treatment and subsequently cured to the point of their blood antigen concentration reducing sufficiently to appear RDT-negative by the time of interview, $p_{\text{cured and reverted}}$, was assigned using the adjustment presented previously.[6] The probability of the success of each individual's antimalarial treatment was derived from treatment failure data compiled by the World Health Organization, matching the type of antimalarial to the country and year in which it was received.[7-8]

In Bayesian hierarchical notation all these layers sitting below the likelihood function may be written as follows:

for i in $1, \ldots, N_{\text{individuals}}$:

$$\text{logit}\, p_{\text{pos.}[i]} = \beta_m^{\text{pos.}} m_i + \beta_a^{\text{pos.}} a_i + \beta_w^{\text{pos.}} w_i + \beta_b^{\text{pos.}} b_i + \text{logit}\, \text{MAP}_{\text{pos.}[i]} + f_{\text{pos.}}(\text{location}_i)$$
$$\text{logit}\, p_{\text{bg}[i]} = \beta_m^{\text{bg}} m_i + \beta_a^{\text{bg}} a_i + \beta_w^{\text{bg}} w_i + \beta_b^{\text{bg}} b_i + \text{logit}\, \text{BRT}_{\text{bg}[i]} + f_{\text{bg}}(\text{location}_i)$$
$$\text{logit}\, p_{\text{treat|maf}[i]} = \beta_m^{\text{treat}} m_i + \beta_a^{\text{treat}} a_i + \beta_w^{\text{treat}} w_i + \beta_b^{\text{treat}} b_i + \text{logit}\, \text{BRT}_{\text{treat}[i]} + f_{\text{treat|maf}}(\text{location}_i)$$
$$\text{logit}\, p_{\text{treat|bg}[i]} = \beta_m^{\text{treat}} m_i + \beta_a^{\text{treat}} a_i + \beta_w^{\text{treat}} w_i + \beta_b^{\text{treat}} b_i + \text{logit}\, \text{BRT}_{\text{treat}[i]} + f_{\text{treat|bg}}(\text{location}_i)$$
$$\text{logit}\, p_{\text{treat|both}[i]} = \beta_m^{\text{treat}} m_i + \beta_a^{\text{treat}} a_i + \beta_w^{\text{treat}} w_i + \beta_b^{\text{treat}} b_i + \text{logit}\, \text{BRT}_{\text{treat}[i]} + f_{\text{treat|both}}(\text{location}_i)$$
$$\text{logit}\, \text{maf}_{[i]} = \alpha_0 + \alpha_1\, p_{\text{pos.}[i]} + \alpha_2 \left(p_{\text{pos.}[i]}\right)^2$$

for j in $1, \ldots, N_{\text{surveys}}$:

$$\text{logit}\, p_{\text{prick[j]}} \sim \text{Normal}\left(\mu_{\text{prick}}, \sigma^2_{\text{prick}}\right)$$
$$\text{logit}\, p_{\text{undertreat[j]}} \sim \text{Normal}\left(\mu_{\text{undertreat}}, \sigma^2_{\text{undertreat}}\right)$$

$$\text{logit}\, p_{\text{overtreat}[j]} \sim \text{Normal}(\mu_{\text{overtreat}}, \sigma^2_{\text{overtreat}})$$
$$\text{logit}\, p_{\text{presumptive}[j]} \sim \text{Normal}(\mu_{\text{presumptive}}, \sigma^2_{\text{presumptive}})$$

$$f_{\text{pos.}}(\cdot) \sim \text{Gaussian Process}[\theta_{\text{pos.}}]$$
$$f_{\text{bg}}(\cdot) \sim \text{Gaussian Process}[\theta_{\text{bg}}]$$
$$f_{\text{treat|maf}}(\cdot) \sim \text{Gaussian Process}[\theta_{\text{treat|maf}}]$$
$$f_{\text{treat|bg}}(\cdot) \sim \text{Gaussian Process}[\theta_{\text{treat|bg}}]$$
$$f_{\text{treat|both}}(\cdot) \sim \text{Gaussian Process}[\theta_{\text{treat|both}}]$$

All free parameters and hyper-parameters are assigned suitable priors (details may be found in the TMB model code available on GitHub[20]).

The top layer of the hierarchical model corresponds to the categorical likelihood for each category form by an individual's observed RDT test result (P: 0 = negative, 1 = positive), caregiver-reported fever status within the past 14 days (F: 0 = no, 1 = yes), treatment sought response (T: 0 = no, 1 = yes), heel-prick conducted response (H: 0 = no, 1 = yes, 2 = missing data), and antimalarials received response (A: 0 = no, 1 = yes).

The probability of each category was found by enumerating each possible scenario whereby an individual could end up in that category. These are written mathematically below with explanatory notes. Calculations are omitted for cases with unknown heel-prick status for brevity: these are simply compiled by summation of the equivalent probabilities for the remaining outcomes with and without a heel-prick, weighted by the probability of having or not having received a heel-prick.

$\boldsymbol{P = 1, F = 0}$:

$$p_{\text{pos}[i]} \times (1 - \text{maf}_{[i]}) \times (1 - p_{\text{bg}[i]})$$

The patient has an RDT-patent parasite load but has had neither a causal nor background fever in the past 14 days.

$\boldsymbol{P = 1, F = 1, T = 1, H = 1, A = 1}$:

$$p_{\text{pos}[i]} \times \big(\text{maf}_{[i]} \times (1 - p_{\text{bg}[i]}) \times p_{\text{treat|maf}[i]} + (1 - \text{maf}_{[i]}) \times p_{\text{bg}[i]} \times p_{\text{treat|bg}[i]} + \text{maf}_{[i]} \times p_{\text{bg}[i]} \times p_{\text{treat|both}[i]}\big) \times p_{\text{prick}[\text{survey}[i]]} \times (1 - p_{\text{undertreat}[\text{survey}[i]]}) \times (1 - p_{\text{cured and reverted}[i]})$$

The patient has one of the three possible fever types and has sought treatment accordingly; at treatment they received a heel-prick, were not explicitly under-treated (i.e. received antimalarials), but had not cleared their parasite load and reverted to RDT negative by the time of survey.

$\boldsymbol{P = 0, F = 1, T = 1, H = 1, A = 1}$:

$$(1 - p_{\text{pos}[i]}) \times p_{\text{bg}[i]} \times p_{\text{treat|bg}[i]} \times p_{\text{prick}[\text{survey}[i]]} \times p_{\text{overtreat}[\text{survey}[i]]} + p_{\text{pos}[i]} \times \big(\text{maf}_{[i]} \times (1 - p_{\text{bg}[i]}) \times p_{\text{treat|maf}[i]} + (1 - \text{maf}_{[i]}) \times p_{\text{bg}[i]} \times p_{\text{treat|bg}[i]} + \text{maf}_{[i]} \times p_{\text{bg}[i]} \times p_{\text{treat|both}[i]}\big) \times p_{\text{prick}[\text{survey}[i]]} \times \big(1 - p_{\text{undertreat}[\text{survey}[i]]}\big) \times p_{\text{cured and reverted}[i]}$$

Either the patient followed the course of events in the scenario above but *did* successfully clear their parasite load and revert to RDT negative by the time of survey; *or*, they had only a background fever, received a heel-prick that presumably returned a negative result but were given antimalarials anyway (explicit over-treatment).

$\boldsymbol{P=0, F=1, T=1, H=0, A=1}$:
$$\left(1-p_{\mathrm{pos}[i]}\right) \times p_{\mathrm{bg}[i]} \times p_{\mathrm{treat|bg}[i]} \times \left(1-p_{\mathrm{prick}[\mathrm{survey}[i]]}\right) \times p_{\mathrm{presumptive}[\mathrm{survey}[i]]} + p_{\mathrm{pos}[i]} \times \left(\mathrm{maf}_{[i]} \times \left(1-p_{\mathrm{bg}[i]}\right) \times p_{\mathrm{treat|maf}[i]} + \left(1-\mathrm{maf}_{[i]}\right) \times p_{\mathrm{bg}[i]} \times p_{\mathrm{treat|bg}[i]} + \mathrm{maf}_{[i]} \times p_{\mathrm{bg}[i]} \times p_{\mathrm{treat|both}[i]}\right) \times \left(1-p_{\mathrm{prick}[\mathrm{survey}[i]]}\right) \times p_{\mathrm{presumptive}[\mathrm{survey}[i]]} \times p_{\text{cured and reverted}[i]}$$

The same range of possibilities as above except that since no heel prick was given this is considered a presumptive treatment event; whether or not that treatment could be considered implicit over-treatment or not of course depends on whether or not they did indeed have RDT-patent parasitemia at the point of treatment (to which a probability is assigned, although the definitive outcome is unknown).

$\boldsymbol{P=1, F=1, T=1, H=1, A=0}$:
$$p_{\mathrm{pos}[i]} \times \left(\mathrm{maf}_{[i]} \times \left(1-p_{\mathrm{bg}[i]}\right) \times p_{\mathrm{treat|maf}[i]} + \left(1-\mathrm{maf}_{[i]}\right) \times p_{\mathrm{bg}[i]} \times p_{\mathrm{treat|bg}[i]} + \mathrm{maf}_{[i]} \times p_{\mathrm{bg}[i]} \times p_{\mathrm{treat|both}[i]}\right) \times p_{\mathrm{prick}[\mathrm{survey}[i]]} \times p_{\mathrm{undertreat}[\mathrm{survey}[i]]}$$

Here it is assumed that the probability of a patient becoming parasite positive in between seeking treatment for their fever in the past 14 days and the time of survey is negligible; therefore the patient must have been parasite positive at time of seeking treatment so failure to receive antimalarials counts as an explicit under-treatment.

$\boldsymbol{P=1, F=1, T=1, H=0, A=1}$:
$$p_{\mathrm{pos}[i]} \times \left(\mathrm{maf}_{[i]} \times \left(1-p_{\mathrm{bg}[i]}\right) \times p_{\mathrm{treat|maf}[i]} + \left(1-\mathrm{maf}_{[i]}\right) \times p_{\mathrm{bg}[i]} \times p_{\mathrm{treat|bg}[i]} + \mathrm{maf}_{[i]} \times p_{\mathrm{bg}[i]} \times p_{\mathrm{treat|both}[i]}\right) \times \left(1-p_{\mathrm{prick}[\mathrm{survey}[i]]}\right) \times p_{\mathrm{presumptive}[\mathrm{survey}[i]]} \times \left(1-p_{\text{cured and reverted}[i]}\right)$$

Since no heel-prick test was conducted but antimalarials were given this is considered a case of presumptive treatment.

$\boldsymbol{P=1, F=1, T=1, H=0, A=0}$:
$$p_{\mathrm{pos}[i]} \times \left(\mathrm{maf}_{[i]} \times \left(1-p_{\mathrm{bg}[i]}\right) \times p_{\mathrm{treat|maf}[i]} + \left(1-\mathrm{maf}_{[i]}\right) \times p_{\mathrm{bg}[i]} \times p_{\mathrm{treat|bg}[i]} + \mathrm{maf}_{[i]} \times p_{\mathrm{bg}[i]} \times p_{\mathrm{treat|both}[i]}\right) \times \left(1-p_{\mathrm{prick}[\mathrm{survey}[i]]}\right) \times \left(1-p_{\mathrm{presumptive}[\mathrm{survey}[i]]}\right)$$

No heel-prick test was conducted and no antimalarials were given; since we again assume that the probability of the patient acquiring their parasite positive status after seeking treatment but before the survey, this will be counted later as a case of (implicit) under-treatment (a missed opportunity).

$\boldsymbol{P=1, F=1, T=0}$:
$$p_{\mathrm{pos}[i]} \times \left(\mathrm{maf}_{[i]} \times \left(1-p_{\mathrm{bg}[i]}\right) \times \left(1-p_{\mathrm{treat|maf}[i]}\right) + \left(1-\mathrm{maf}_{[i]}\right) \times p_{\mathrm{bg}[i]} \times \left(1-p_{\mathrm{treat|bg}[i]}\right) + \mathrm{maf}_{[i]} \times p_{\mathrm{bg}[i]} \times \left(1-p_{\mathrm{treat|both}[i]}\right)\right)$$

The patient has one of the three fever types but has not sought treatment.

$\boldsymbol{P=0, F=1, T=1, H=1, A=0}$:

$\left(1-p_{\mathrm{pos}[i]}\right) \times p_{\mathrm{bg}[i]} \times p_{\mathrm{treat|bg}[i]} \times p_{\mathrm{prick}[\mathrm{survey}[i]]} \times (1-p_{\mathrm{overtreat}[\mathrm{survey}[i]]})$

Since the patient experienced a fever but has no patent parasite load and did not receive antimalarials it is inferred that they must have had a background fever; in this case they were given a heel-prick and not inappropriately treated with antimalarials (explicit over-treatment).

$\boldsymbol{P=0, F=1, T=1, H=0, A=0}$:

$\left(1-p_{\mathrm{pos}[i]}\right) \times p_{\mathrm{bg}[i]} \times p_{\mathrm{treat|bg}[i]} \times (1-p_{\mathrm{prick}[\mathrm{survey}[i]]}) \times (1-p_{\mathrm{presumptive}[\mathrm{survey}[i]]})$

Since the patient experienced a fever but has no RDT-patent parasite load and did not receive antimalarials it is again inferred that they must have had a background fever; in this case they were not given a heel-prick but were also not presumptively (over-)treated.

$\boldsymbol{P=0, F=1, T=0}$:

$\left(1-p_{\mathrm{pos}[i]}\right) \times p_{\mathrm{bg}[i]} \times (1-p_{\mathrm{treat|bg}[i]})$

Since the individual experienced a fever but has no patent parasite load and did not seek treatment (nor therefore receive antimalarials) it is again inferred that they must have had a background fever.

$\boldsymbol{P=0, F=0}$:

$\left(1-p_{\mathrm{pos}[i]}\right) \times (1-p_{\mathrm{bg}[i]})$

The individual had neither a patent parasite load, nor a background fever during the past 14 days.

Once this model was coded in TMB and fit against the observed data, it was possible to arithmetically compute the posterior probabilities of each individual's fever type, though the diversity of possible observed categories made this is a formidable book-keeping exercise. The full set of calculations can be seen in the publicly available R script[20] but one case is explained below to illustrate the procedure.

$\boldsymbol{P=1, F=1, T=1, H=1, A=1}$:

To compute the probability that an individual in this category had a malaria-attributable fever (with no background fever) this component of the class probability is divided by the sum of all components.

$P(\mathrm{maf\ only})[\mathrm{i}] = \left(\mathrm{maf}_{[i]} \times \left(1-p_{\mathrm{bg}[i]}\right) \times p_{\mathrm{treat|maf}[i]}\right) / \left(\mathrm{maf}_{[i]} \times \left(1-p_{\mathrm{bg}[i]}\right) \times p_{\mathrm{treat|maf}[i]} + (1-\mathrm{maf}_{[i]}) \times p_{\mathrm{bg}[i]} \times p_{\mathrm{treat|bg}[i]} + \mathrm{maf}_{[i]} \times p_{\mathrm{bg}[i]} \times p_{\mathrm{treat|both}[i]}\right)$

In this calculation it can be seen that the posterior probability assigned to the individual's fever type depends on the relative probabilities that they would have each type of fever *and* the conditional probabilities that they would seek treatment. Since individual-level covariates and random field models inform both these calculations, the model is both tailored to each individual's circumstances and borrows strength from the observed behaviour of their neighbours. When these fever type probabilities were summed to produce survey-level estimates (e.g. of the relative fraction of malarial and non-malarial fevers amongst malaria-positive individuals), the accompanying design weights were used as supplied within the DHS Program surveys. The surveys were designed so that the stratification applied to the sampling relative to the total population was encapsulated in these design weights such that nationally-representative estimates were recovered upon application of the weights.

References

1 Dalrymple U, Arambepola R, Gething PW, Cameron E. How long do rapid diagnostic tests remain positive after anti-malarial treatment? *Malar J* 2018; **17**. DOI:10.1186/s12936-018-2371-9.

2 Dalrymple U, Cameron E, Bhatt S, Weiss DJ, Gupta S, Gething PW. Quantifying the contribution of *Plasmodium falciparum* malaria to febrile illness amongst African children. *Elife* 2017; **6**: e29198

3 Maire N, Smith T, Ross A, Owusu-agyei S, Dietz K, Molineaux L. A model for natural immunity to asexual blood stages of *Plasmodium falciparum* malaria in endemic areas. *Am J Trop Med Hyg* 2006; **75**: 19–31.

4 Smith T, Ross A, Maire N, Rogier C, Trape J-F, Molineaux L. An epidemiologic model of the incidence of acute illness in *Plasmodium falciparum* malaria. *Am J Trop Med Hyg* 2006; **75**: 56–62.

5 Ross A, Maire N, Molineaux L, Smith T. An epidemiologic model of severe morbidity and mortality caused by *Plasmodium falciparum*. *Am J Trop Med Hyg* 2006; **75**: 63–73.

6 World Health Organization. Global database on antimalarial drug efficacy and resistance. 2018.

7 World Health Organization. Global Report on Antimalarial Drug Efficacy and Drug Resistance 2000-2010. 2010.

8 Dalrymple U, Cameron E. MAF-NMFI GitHub: udalrymple. 2018. https://github.com/udalrymple/MAF-NMFI
