# Supplementary material for: The contribution of non-malarial febrile illness co-infections to Plasmodium falciparum case counts in health facilities in sub-Saharan Africa
Source: Malar J. 2019 Jun 11;18:195. doi: 10.1186/s12936-019-2830-y (PMC6560910; doi:10.1186/s12936-019-2830-y)
Supplement: Supplementary file 7 — Additional file 7: Figure S1. (a) Estimates of proportion of fevers amongst malaria-positive presenting to public health clinics that are causally due to MAF (red sections), NMFI accompanied by an asymptomatic P. falciparum malaria infection (blue sections) and co-symptomatic MAF and NMFI (yellow sections); and (b) as (a), also including NMFI not accompanied by a P. falciparum malaria infection (grey sections); amongst febrile children under 5 years of age presenting to public health clinics amongst children from household surveys in 41 country-years in sub-Saharan Africa, 2006–2016. [file 12936_2019_2830_MOESM7_ESM.pdf]

Additional File 7

Additional Figure 1a) Estimates of proportion of fevers amongst malaria-positive presenting to public health clinics that are causally due to MAF (red sections), NMFI accompanied by an asymptomatic *P. falciparum* malaria infection (blue sections) and co-symptomatic MAF and NMFI (yellow sections); and Additional Figure1 b) as (a), also including NMFI not accompanied by a *P. falciparum* malaria infection (grey sections); amongst febrile children under 5 years of age presenting to public health clinics amongst children from household surveys in 41 country-years in sub-Saharan Africa, 2006-2016.

Additional Figure 1 (a)

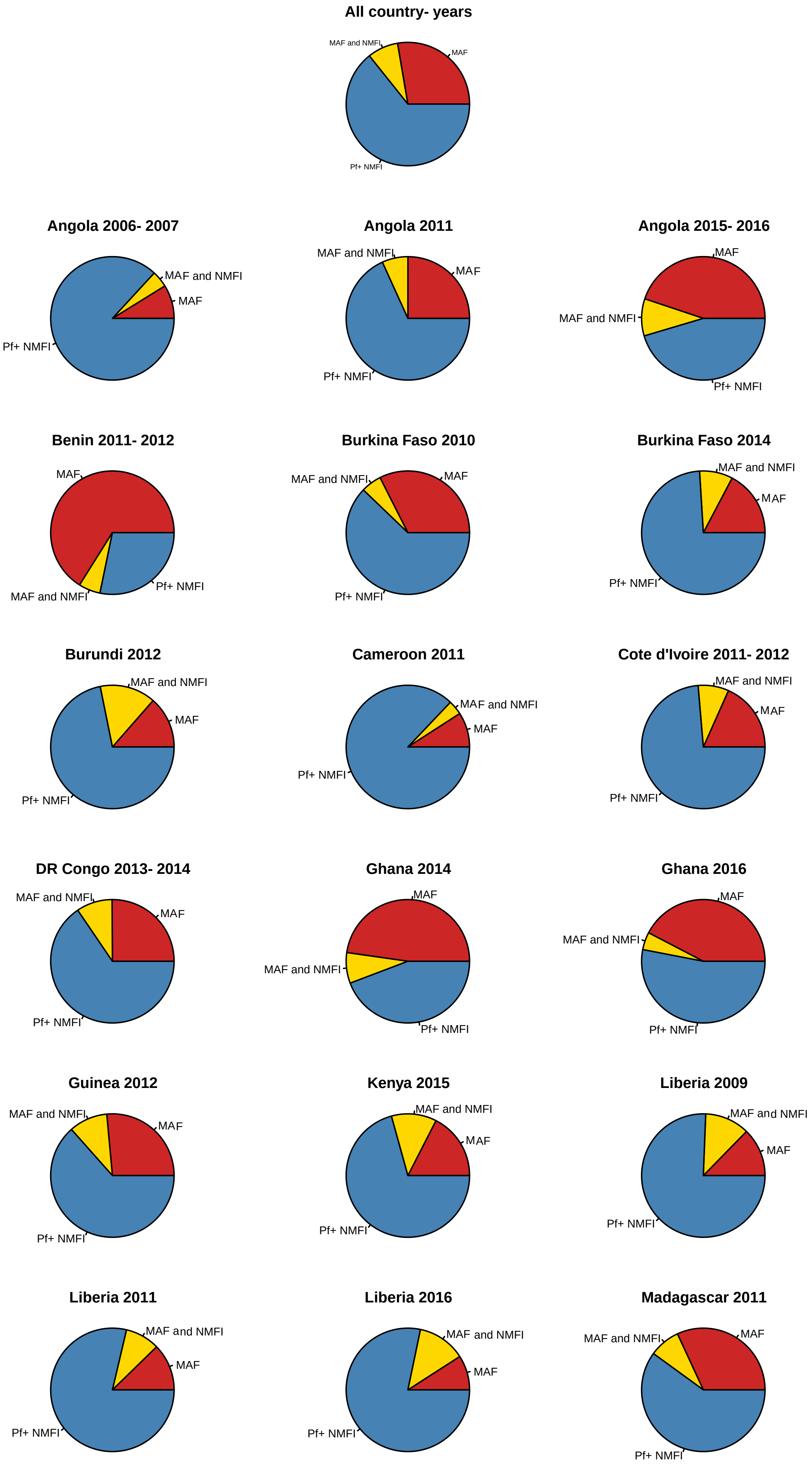

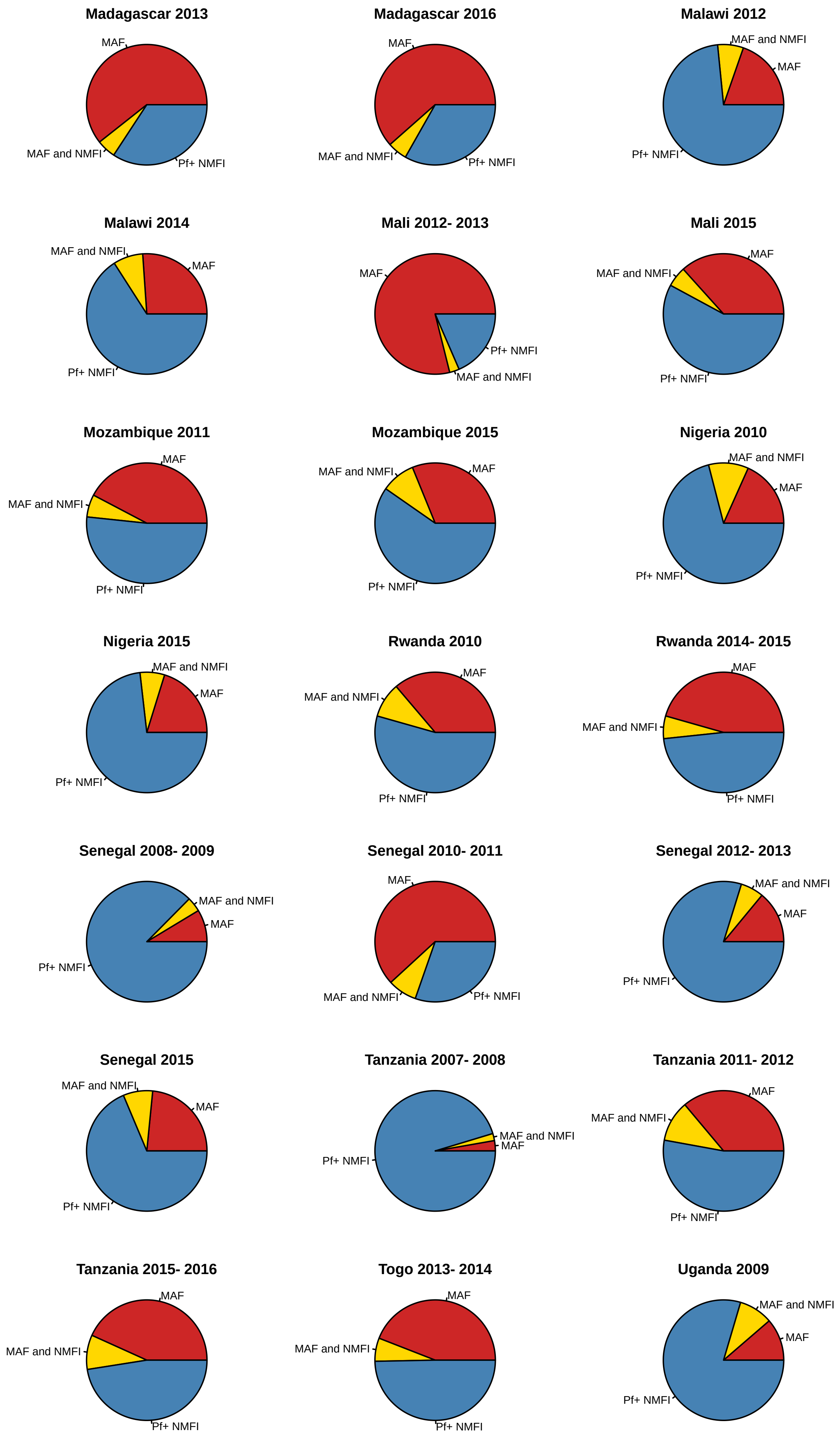
Madagascar 2013
MAF
MAF and NMFI
Pf+ NMFI
Madagascar 2016
MAF
MAF and NMFI
Pf+ NMFI
Malawi 2012
MAF and NMFI
MAF
Pf+ NMFI
Malawi 2014
MAF and NMFI
MAF
Pf+ NMFI
Mali 2012- 2013
MAF
Pf+ NMFI
MAF and NMFI
Mali 2015
MAF
MAF and NMFI
Pf+ NMFI
Mozambique 2011
MAF
MAF and NMFI
Pf+ NMFI
Mozambique 2015
MAF and NMFI
MAF
Pf+ NMFI
Nigeria 2010
MAF and NMFI
MAF
Pf+ NMFI
Nigeria 2015
MAF and NMFI
MAF
Pf+ NMFI
Rwanda 2010
MAF
MAF and NMFI
Pf+ NMFI
Rwanda 2014- 2015
MAF
MAF and NMFI
Pf+ NMFI
Senegal 2008- 2009
MAF and NMFI
MAF
Pf+ NMFI
Senegal 2010- 2011
MAF
MAF and NMFI
Pf+ NMFI
Senegal 2012- 2013
MAF and NMFI
MAF
Pf+ NMFI
Senegal 2015
MAF and NMFI
MAF
Pf+ NMFI
Tanzania 2007- 2008
MAF and NMFI
MAF
Pf+ NMFI
Tanzania 2011- 2012
MAF
MAF and NMFI
Pf+ NMFI
Tanzania 2015- 2016
MAF
MAF and NMFI
Pf+ NMFI
Togo 2013- 2014
MAF
MAF and NMFI
Pf+ NMFI
Uganda 2009
MAF and NMFI
MAF
Pf+ NMFI

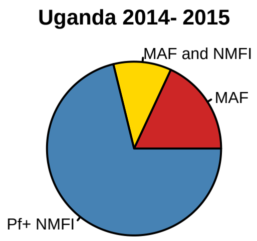
Uganda 2014- 2015
MAF and NMFI
MAF
Pf+ NMFI

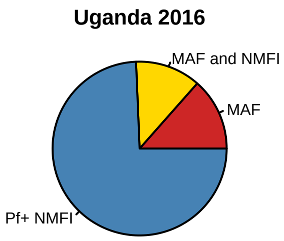
Uganda 2016
MAF and NMFI
MAF
Pf+ NMFI

Additional Figure 1 (b)

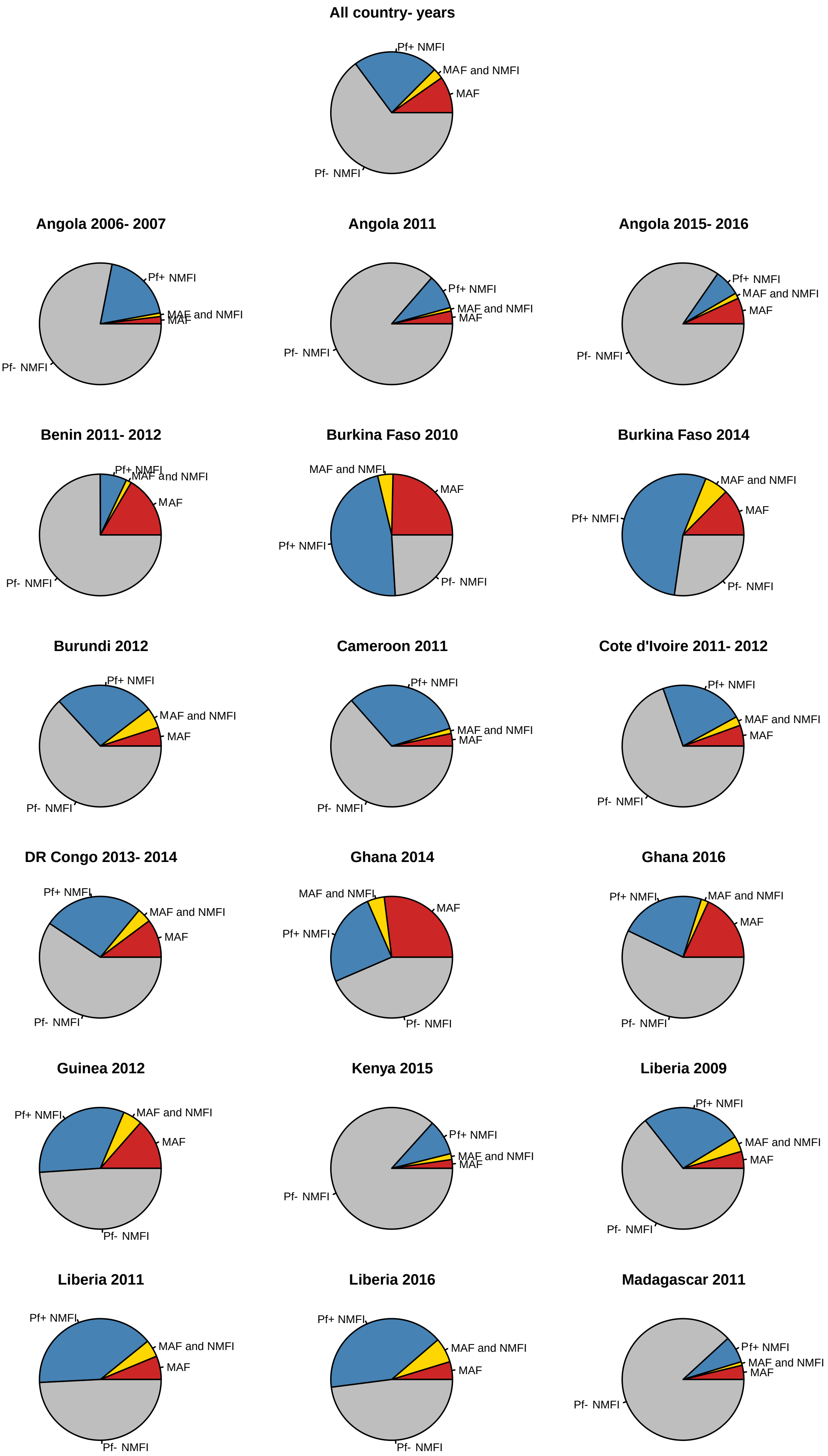

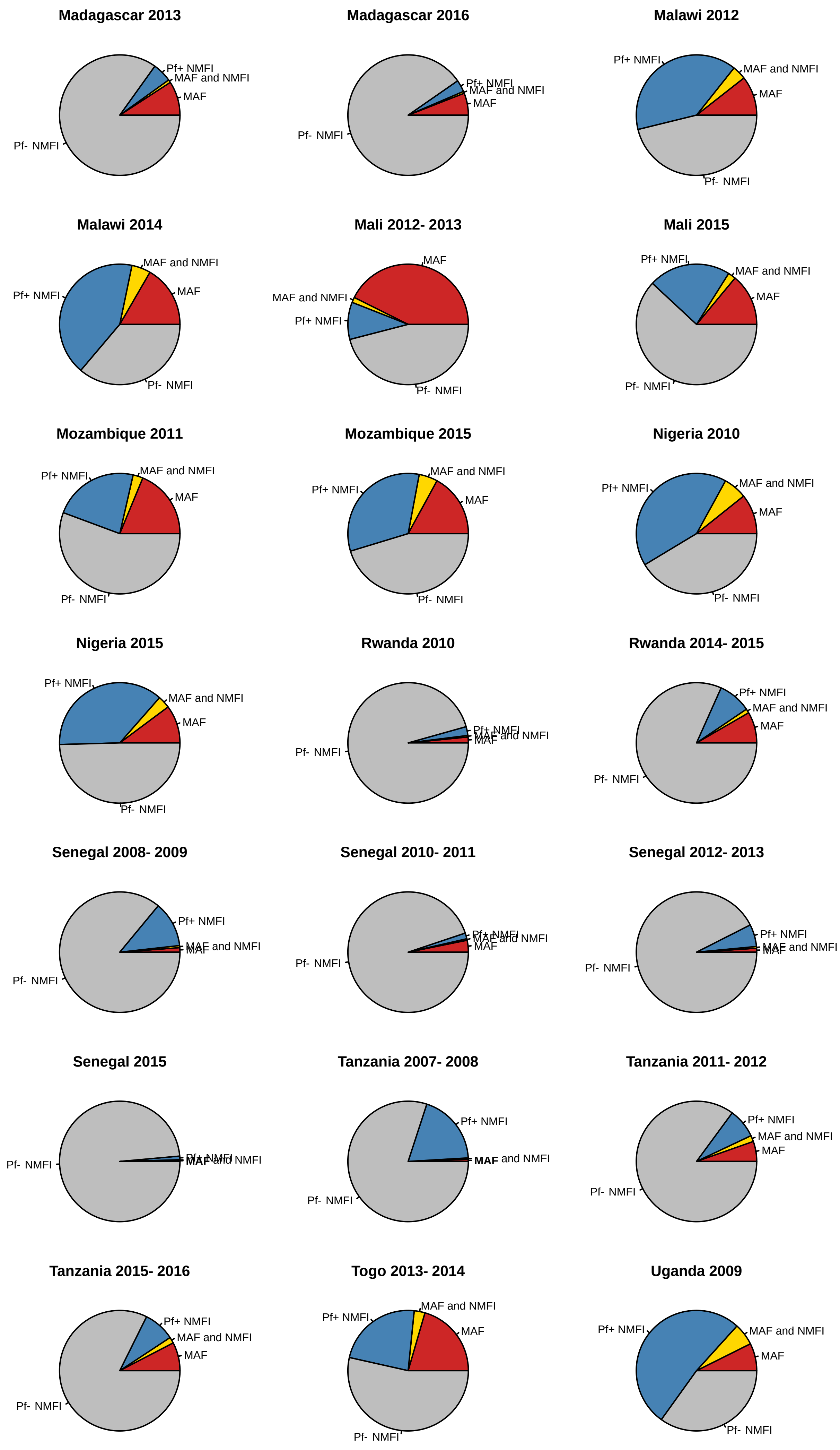

Madagascar 2013
Pf+ NMFI
MAF and NMFI
MAF
Pf- NMFI
Madagascar 2016
Pf+ NMFI
MAF and NMFI
MAF
Pf- NMFI
Malawi 2012
Pf+ NMFI
MAF and NMFI
MAF
Pf- NMFI
Malawi 2014
MAF and NMFI
Pf+ NMFI
MAF
Pf- NMFI
Mali 2012- 2013
MAF
MAF and NMFI
Pf+ NMFI
Pf- NMFI
Mali 2015
Pf+ NMFI
MAF and NMFI
MAF
Pf- NMFI
Mozambique 2011
Pf+ NMFI
MAF and NMFI
MAF
Pf- NMFI
Mozambique 2015
MAF and NMFI
Pf+ NMFI
MAF
Pf- NMFI
Nigeria 2010
MAF and NMFI
Pf+ NMFI
MAF
Pf- NMFI
Nigeria 2015
Pf+ NMFI
MAF and NMFI
MAF
Pf- NMFI
Rwanda 2010
Pf+ NMFI
MAF and NMFI
MAF
Pf- NMFI
Rwanda 2014- 2015
Pf+ NMFI
MAF and NMFI
MAF
Pf- NMFI
Senegal 2008- 2009
Pf+ NMFI
MAF and NMFI
MAF
Pf- NMFI
Senegal 2010- 2011
Pf+ NMFI
MAF and NMFI
MAF
Pf- NMFI
Senegal 2012- 2013
Pf+ NMFI
MAF and NMFI
MAF
Pf- NMFI
Senegal 2015
Pf+ NMFI
MAF and NMFI
MAF
Pf- NMFI
Tanzania 2007- 2008
Pf+ NMFI
MAF and NMFI
Pf- NMFI
Tanzania 2011- 2012
Pf+ NMFI
MAF and NMFI
MAF
Pf- NMFI
Tanzania 2015- 2016
Pf+ NMFI
MAF and NMFI
MAF
Pf- NMFI
Togo 2013- 2014
MAF and NMFI
Pf+ NMFI
MAF
Pf- NMFI
Uganda 2009
Pf+ NMFI
MAF and NMFI
MAF
Pf- NMFI

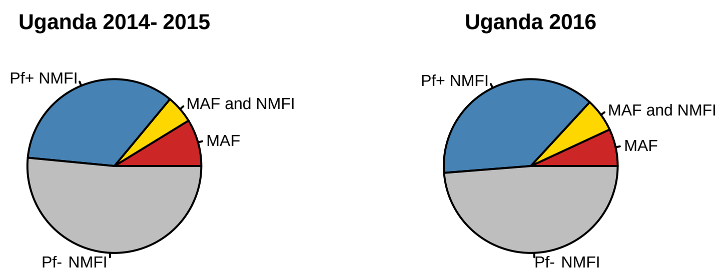
Uganda 2014- 2015
Pf+ NMFI
MAF and NMFI
MAF
Pf- NMFI
Uganda 2016
Pf+ NMFI
MAF and NMFI
MAF
Pf- NMFI
