# Supplementary material for: The contribution of non-malarial febrile illness co-infections to Plasmodium falciparum case counts in health facilities in sub-Saharan Africa
Source: Malar J. 2019 Jun 11;18:195. doi: 10.1186/s12936-019-2830-y (PMC6560910; doi:10.1186/s12936-019-2830-y)
Supplement: Supplementary file 8 — Additional file 8. Proportion of each type of fever presenting at public health clinics in each survey, with associated 95% credible intervals. MAF = malaria-attributable fever; NMFI = non-malarial febrile illness; Pf+/Pf− = P. falciparum RDT positive or negative (at time of seeking treatment). “MAF and NMFI” indicates febrile children with co-symptomatic MAF and NMFI. [file 12936_2019_2830_MOESM8_ESM.pdf]

Additional File 8. Proportion of each type of fever presenting at public health clinics in each survey, with associated 95% credible intervals. MAF= malaria-attributable fever; NMFI = non-malarial febrile illness; Pf+/Pf- = *P. falciparum* RDT positive or negative (at time of seeking treatment). "MAF and NMFI" indicates febrile children with co-symptomatic MAF and NMFI.

Survey	MAF Lower CrI	MAF Median	MAF Upper CrI	MAF and NMFI Lower CrI	MAF and NMFI Median	MAF and NMFI Upper CrI	NMFI Pf+ Lower CrI	NMFI Pf+ Median	NMFI Pf+ Upper CrI	NMFI Pf- Lower CrI	NMFI Pf- Median	NMFI Pf- Upper CrI
**Angola 2006-2007**	0.0037	0.0192	0.0699	0.0011	0.0094	0.0372	0.1508	0.1895	0.2095	0.7644	0.7782	0.7817
**Angola 2011**	0.0197	0.0340	0.0463	0.0012	0.0092	0.0212	0.0849	0.0930	0.1029	0.8619	0.8630	0.8641
**Angola 2015-2016**	0.0466	0.0691	0.0884	0.0027	0.0150	0.0284	0.0604	0.0699	0.0818	0.8406	0.8471	0.8518
**Benin 2011-2012**	0.1325	0.1654	0.2006	0.0025	0.0142	0.0258	0.0566	0.0707	0.0886	0.7331	0.7494	0.7628
**Burkina Faso 2010**	0.2092	0.2466	0.2883	0.0151	0.0408	0.0578	0.4392	0.4723	0.5010	0.2377	0.2406	0.2432
**Burkina Faso 2014**	0.1006	0.1258	0.1491	0.0404	0.0634	0.0824	0.5176	0.5391	0.5563	0.2707	0.2732	0.2751
**Burundi 2012**	0.0328	0.0500	0.0670	0.0280	0.0539	0.0636	0.2521	0.2649	0.2805	0.6306	0.6327	0.6347
**Cameroon 2011**	0.0063	0.0330	0.1166	0.0016	0.0136	0.0767	0.2334	0.3150	0.3487	0.6146	0.6291	0.6325
**Cote d'Ivoire 2011-2012**	0.0302	0.0554	0.0896	0.0028	0.0243	0.0522	0.2038	0.2229	0.2437	0.6912	0.6955	0.6989
**DR Congo 2013-2014**	0.0860	0.1020	0.1197	0.0241	0.0382	0.0504	0.2537	0.2661	0.2781	0.5909	0.5932	0.5951
**Ghana 2014**	0.2373	0.2699	0.3023	0.0254	0.0454	0.0542	0.2301	0.2500	0.2710	0.4268	0.4355	0.4439
**Ghana 2016**	0.1287	0.1816	0.2212	0.0010	0.0197	0.0504	0.2048	0.2274	0.2544	0.5630	0.5716	0.5783
**Guinea 2012**	0.0948	0.1353	0.1755	0.0160	0.0516	0.0852	0.2888	0.3243	0.3552	0.4844	0.4900	0.4941
**Kenya 2015**	0.0102	0.0233	0.0319	0.0039	0.0158	0.0239	0.0878	0.0945	0.1014	0.8652	0.8673	0.8690
**Liberia 2009**	0.0303	0.0453	0.0566	0.0265	0.0418	0.0482	0.2616	0.2699	0.2787	0.6427	0.6451	0.6470
**Liberia 2011**	0.0473	0.0626	0.0740	0.0321	0.0459	0.0575	0.3890	0.4002	0.4119	0.4914	0.4928	0.4942
**Liberia 2016**	0.0226	0.0474	0.0832	0.0274	0.0659	0.0814	0.3913	0.4084	0.4255	0.4796	0.4807	0.4818
**Madagascar 2011**	0.0221	0.0376	0.0575	0.0011	0.0095	0.0238	0.0590	0.0708	0.0830	0.8787	0.8803	0.8813
**Madagascar 2013**	0.0750	0.0916	0.1085	0.0017	0.0077	0.0166	0.0422	0.0517	0.0638	0.8417	0.8487	0.8535
**Madagascar 2016**	0.0487	0.0594	0.0704	0.0007	0.0051	0.0101	0.0251	0.0321	0.0400	0.8996	0.9036	0.9060
**Malawi 2012**	0.0761	0.1060	0.1379	0.0153	0.0373	0.0582	0.3685	0.3959	0.4180	0.4607	0.4625	0.4637
**Malawi 2014**	0.1218	0.1669	0.2021	0.0195	0.0512	0.0761	0.3956	0.4218	0.4502	0.3611	0.3628	0.3641
**Mali 2012-2013**	0.3929	0.4264	0.4512	0.0018	0.0141	0.0325	0.0840	0.0999	0.1192	0.4520	0.4597	0.4664
**Mali 2015**	0.1092	0.1393	0.1645	0.0056	0.0207	0.0514	0.2054	0.2198	0.2370	0.6120	0.6176	0.6229
**Mozambique 2011**	0.1591	0.1876	0.2137	0.0094	0.0268	0.0439	0.2121	0.2292	0.2484	0.5469	0.5567	0.5647
**Mozambique 2015**	0.1409	0.1707	0.2009	0.0183	0.0501	0.0669	0.3052	0.3269	0.3459	0.4465	0.4537	0.4591
**Nigeria 2010**	0.0969	0.1073	0.1159	0.0557	0.0629	0.0670	0.4051	0.4162	0.4282	0.4129	0.4140	0.4151

Additional File 8 (Continued)

Survey	MAF Lower CrI	MAF Median	MAF Upper CrI	MAF and NMFI Lower CrI	MAF and NMFI Median	MAF and NMFI Upper CrI	NMFI Pf+ Lower CrI	NMFI Pf+ Median	NMFI Pf+ Upper CrI	NMFI Pf- Lower CrI	NMFI Pf- Median	NMFI Pf- Upper CrI
**Nigeria 2015**	0.0872	0.1020	0.1149	0.0225	0.0333	0.0446	0.3580	0.3693	0.3820	0.4949	0.4960	0.4970
**Rwanda 2010**	0.0084	0.0158	0.0198	0.0008	0.0041	0.0062	0.0206	0.0237	0.0291	0.9560	0.9569	0.9579
**Rwanda 2014-2015**	0.0670	0.0835	0.0958	0.0011	0.0111	0.0196	0.0807	0.0886	0.0979	0.8163	0.8177	0.8186
**Senegal 2008-2009**	0.0019	0.0120	0.0470	0.0006	0.0055	0.0312	0.0906	0.1218	0.1376	0.8563	0.8568	0.8570
**Senegal 2010-2011**	0.0241	0.0318	0.0378	0.0003	0.0040	0.0087	0.0131	0.0156	0.0192	0.9453	0.9487	0.9512
**Senegal 2012-2013**	0.0017	0.0104	0.0409	0.0004	0.0046	0.0270	0.0341	0.0594	0.0731	0.9201	0.9215	0.9217
**Senegal 2015**	0.0005	0.0033	0.0111	0.0001	0.0011	0.0069	0.0040	0.0097	0.0137	0.9838	0.9847	0.9849
**Tanzania 2007-2008**	0.0013	0.0055	0.0192	0.0006	0.0038	0.0162	0.1773	0.1903	0.1961	0.7971	0.7993	0.8003
**Tanzania 2011-2012**	0.0426	0.0539	0.0621	0.0073	0.0164	0.0201	0.0738	0.0789	0.0852	0.8490	0.8517	0.8541
**Tanzania 2015-2016**	0.0682	0.0765	0.0828	0.0089	0.0163	0.0185	0.0787	0.0840	0.0892	0.8210	0.8244	0.8273
**Togo 2013-2014**	0.1614	0.2052	0.2387	0.0051	0.0290	0.0630	0.2061	0.2314	0.2578	0.5269	0.5358	0.5440
**Uganda 2009**	0.0631	0.0738	0.0819	0.0457	0.0594	0.0646	0.5088	0.5187	0.5299	0.3486	0.3497	0.3510
**Uganda 2014-2015**	0.0705	0.0878	0.0996	0.0282	0.0523	0.0599	0.3356	0.3460	0.3588	0.5155	0.5167	0.5181
**Uganda 2016**	0.0485	0.0694	0.0910	0.0281	0.0625	0.0709	0.3687	0.3822	0.4011	0.4857	0.4890	0.4919
